# A GRFa2/Prop1/Stem (GPS) Cell Niche in the Pituitary

**DOI:** 10.1371/journal.pone.0004815

**Published:** 2009-03-13

**Authors:** Montse Garcia-Lavandeira, Víctor Quereda, Ignacio Flores, Carmen Saez, Esther Diaz-Rodriguez, Miguel A. Japon, Aymee K. Ryan, Maria A. Blasco, Carlos Dieguez, Marcos Malumbres, Clara V. Alvarez

**Affiliations:** 1 Department of Physiology, School of Medicine, University of Santiago de Compostela (USC), Santiago de Compostela, Spain; 2 Cell Division and Cancer Group, Centro Nacional de Investigaciones Oncologicas (CNIO), Madrid, Spain; 3 Telomeres and Telomerase Group, Centro Nacional de Investigaciones Oncológicas (CNIO), Madrid, Spain; 4 Department of Pathology, Hospital Universitario Virgen del Rocio, Seville, Spain; 5 Department of Human Genetics, McGill University (MUHC), Montreal, Quebec, Canada; 6 CIBER Obesity & Nutrition (ISCIII), Santiago de Compostela, Spain; University of Las Palmas de Gran Canaria, Spain

## Abstract

**Background:**

The adult endocrine pituitary is known to host several hormone-producing cells regulating major physiological processes during life. Some candidates to progenitor/stem cells have been proposed. However, not much is known about pituitary cell renewal throughout life and its homeostatic regulation during specific physiological changes, such as puberty or pregnancy, or in pathological conditions such as tumor development.

**Principal Findings:**

We have identified in rodents and humans a niche of non-endocrine cells characterized by the expression of GFRa2, a Ret co-receptor for Neurturin. These cells also express b-Catenin and E-cadherin in an oriented manner suggesting a planar polarity organization for the niche. In addition, cells in the niche uniquely express the pituitary-specific transcription factor Prop1, as well as known progenitor/stem markers such as Sox2, Sox9 and Oct4. Half of these GPS (GFRa2/Prop1/Stem) cells express S-100 whereas surrounding elongated cells in contact with GPS cells express Vimentin. GFRa2+-cells form non-endocrine spheroids in culture. These spheroids can be differentiated to hormone-producing cells or neurons outlining the neuroectoderm potential of these progenitors. In vivo, GPSs cells display slow proliferation after birth, retain BrdU label and show long telomeres in its nuclei, indicating progenitor/stem cell properties in vivo.

**Significance:**

Our results suggest the presence in the adult pituitary of a specific niche of cells characterized by the expression of GFRa2, the pituitary-specific protein Prop1 and stem cell markers. These GPS cells are able to produce different hormone-producing and neuron-like cells and they may therefore contribute to postnatal pituitary homeostasis. Indeed, the relative abundance of GPS numbers is altered in Cdk4-deficient mice, a model of hypopituitarism induced by the lack of this cyclin-dependent kinase. Thus, GPS cells may display functional relevance in the physiological expansion of the pituitary gland throughout life as well as protection from pituitary disease.

## Introduction

The pituitary gland is a central endocrine organ that regulates basic physiological functions such as growth, stress response, reproduction, lactation and metabolic homeostasis. The adenopituitary (AP) hosts several endocrine cell types secreting hormones that regulate the function of other organs and endocrine glands throughout life. Thus, somatotrophs, lactotrophs and thyrotrophs secrete growth hormone (GH), prolactin (PRL), and thyroid-stimulating hormone (TSH) respectively; corticotrophs secrete adrenocorticotropic hormone (ACTH) and gonadotrophs secrete luteinizing hormone (LH) and/or follicle-stimulating hormone (FSH). In addition, some non-hormonal folliculostellate cells have been described whose function is not well understood [1]–[3]. All these cells in the AP arise during development from a common ectodermal primordium known as the Rathke's pouch [4]. However, not much is known on pituitary cell renewal throughout life and its homeostatic regulation during specific physiological changes such as puberty or pregnancy or in pathological conditions such as tumor development. To explain these changes, both cell proliferation of the individual differentiated secretory cells and asymmetric proliferation followed by terminal differentiation of adult stem cells have been proposed [5], [6].

Although the identity of adult pituitary stem cells is not well established, several stem/progenitor cell types have been previously proposed to maintain pituitary homeostasis and generate endocrine cells. A side population (SP) that efficiently excludes the Hoechst 33342 vital dye has been shown to segregate with sphere-forming cells in the pituitary [7]. In addition, pituitary colony-forming cells (PCFCs) that display notable clonogenic potential have also been isolated [8]. However, the only common marker studied for these cells was Sca1 and their position in the pituitary was not well understood [7], [9]. Recently, the presence of Sox2+/Sox9− of the mouse pituitary has been described and proposed to mark stem cells, localized both as an epithelial layer but also heavily intermingled with the differentiated cells [10], while more differentiated progenitors or transit-amplifying cells would become Sox2+/Sox9+. Genetic approaches using transgenic mice expressing GFP under the Nestin promoter identified a population of Nestin+ cells that in vitro behaves as progenitors; however, these cells would only contribute post-puberally to cell-renewal in the adult pituitary [11].

In this manuscript we describe a niche of putative stem cells that express the Glial cell line-derived neurotrophic factor (GDNF) receptor alpha 2 (GFRa2). GFRa2 belongs to a family of receptors (GFRa1-4) that modulate signaling pathways initiated by their ligands, GDNF, Neurturin (NTN), Artemin (ART) and Persephin (PSP). These proteins function as co-receptors of the tyrosine kinase Ret [12]–[14]. GFRa2 functions as an specific NTN receptor as demonstrated in vivo by the almost identical phenotype of mice deficient in either NTN or GFRa2 [15], [16]. In some tissues such as testis and ovary, GFRa1 and 2 receptors are expressed in putative germ-line stem cells [17]–[19]. In the pituitary, somatotrophs (GH) are the only secretory cells expressing Ret and GFRa1 either in rat [20] or in humans [21]. We report here that GFRa2 is expressed in a niche of non-hormonal putative stem/progenitor cells in the pituitary. GFRa2-positive (GFRa2+) cells are organized in a single-cell layer around the cleft originated from the Rathke's pouch. These niche cells display a clear expression of the pituitary specific homeobox protein Prophet of Pit1 (Prop1), a transcription factor required for pituitary development and mutated in pituitary disease [22]–[26]. In addition, these niche cells also express well-established stem cell markers such as Oct4, Sox2, Sox9 and we will refer to them as GPS (**G**FRa2+, **P**rop1+, **S**tem) cells.

## Results

### GFRa2 expression is mostly restricted to a polarized niche in the pituitary

**Figure 1:**
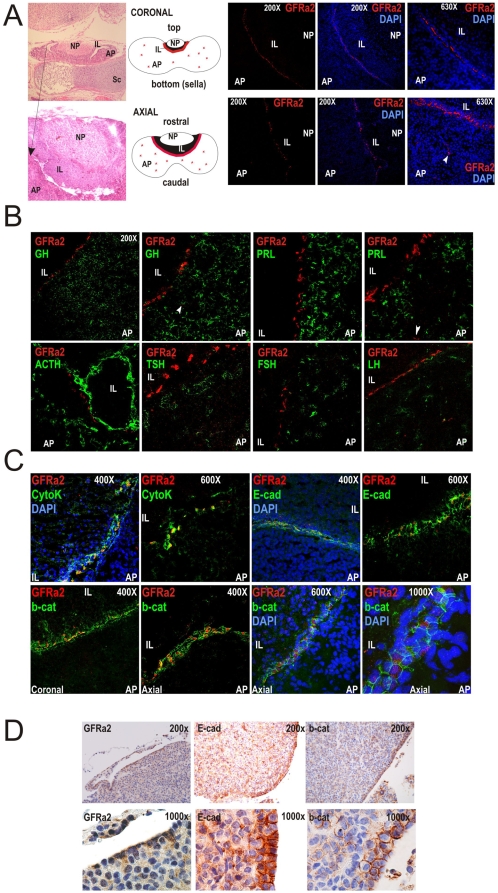
GFRa2+-expressing cells form a line of epithelial non-secretory cells in the adult pituitary of rats and mice. A) Coronal and axial sections stained with Hematoxylin and Eosin (H&E) to show pituitary location under the hypothalamus and on top of the sphenoid sella turcica (Sc) and the disposition of the three pituitary lobes: adenopituitary (AP), intermediate lobe (IL) and neuropituitary (NP) where end-terminals of hypothalamic axons release ADH and Oxytocin. In the rat pituitary, GFRa2+ cells (red) arrange in a precise line in the frontier between the AP and the IL. Very few less intense cells are found dispersed through the AP (arrowhead). B) GFRa2+ cells [either lined or scattered (arrowhead)] do not express any pituitary hormone. C) GFRa2+ cells are epithelial cells with enhanced expression of Cytokeratins, E-cadherin and beta-Catenin. Coronal versus Axial sections demonstrates the orientation of the GFRa2 cells within the niche. In the coronal axis, GFRa2 or b-Catenin appear respectively as a thin line or a U-shaped green staining; in the axial axis GFRa2 appears as a broad surface while b-Catenin shows a ring shaped staining in a perpendicular orientation. D) Localization of GFRa2 cells and co-localization with E-cadherin and b-Catenin in mouse pituitaries.

GFRa2 is expressed in the rat pituitary at similar levels to testis (Figure S1A) or ovary (data not shown), two other endocrine glands where GFRa1 and 2 receptors had been previously described [17]–[19]. In the murine pituitary, GFRa2 expression is restricted to a distinct subset of non-endocrine cells lined to a single-cell layer in the marginal zone (MZ) around the cleft between the intermediate lobe (IL) and the AP (Figure 1). The MZ had been proposed to harbor stem/progenitor cells originated from the Rathke's pouch from which the endocrine cells could be produced but still no clear proof has been found [5], [9]. This layer of cells opposed to the cleft originates, like the AP, from the Rathke's pouch formed from the oral ectoderm during embryonic development. A very limited number of isolated GRFa2+ cells are distributed throughout the AP (Figure 1A). In total, GRFa2+ cells accounts for about 0.9% cells of the adult mouse pituitary (Figure S1B). GFRa2+ cells do not express any pituitary hormone (Figure 1B) but shows a significant expression of epithelial markers such as Cytokeratins and E-cadherin (Figure 1C–D and Table 1). These GFRa2+ cells also display a clear expression of b-Catenin (Figure 1C –rat- and D –mouse-), whose labeling is rarely positive in other cells of this endocrine gland (Figure S1-C). The GFRa2+ niche in the MZ seems to have a Planar Polarity organization. Thus, the anti-GFRa2 antibody stains a very thin line in the coronal axis of the MZ cells. However, GFRa2 stains broadly on MZ cells in the axial axis. Similar polarization is found with b-Catenin where each cell appears as a U-shaped line in coronal sections versus complete rings in axial sections (Figure 1C and Videos S1 and S2). Interestingly, the GFRa2 (membrane) and b-Catenin (cytoplasm) signals are perpendicular (see Axial 400× and 1000× sections), suggesting that the GFRa2 cell niche is formed of cylindrical cells with Planar Polarity coordination, a specific coordination of an epithelial layer of cells to behave with a physiological direction (recently reviewed in [27].

**Table 1:** Markers expressed by GFRa2+ cells.

Marker	% of GFRa2+ cells containing the marker*
**Hormones**	
GH	0%
ACTH	0%
PRL	0%
TSH	0%
FSH	0%
LH	0%
**Epithelial markers and Wnt pathway**	
Multi-Cytokeratin	90%
E-cadherin	86%
b-Catenin	91%
**GFRa2 pathway**	
Ret	78%
GFRa1	0%
NTN	0%
**Stem cell markers**	
SSEA4	100%
Prop1	99%
Oct4	94%
Sox2	91%
Sox9	96%
Sox4	0%
Nestin	0%
Nanog	0%
Isl-1	0%
Pax6	0%
**Other Markers**	
S-100	43%
Vimentin	<3%
**Proliferation markers**	
Ki67 in adult GPS	0%
Ki67 at 10 days	13%

* Percentages are calculated counting cells from (confocal) microscopy pictures (magnification higher than 400×). For each combination of markers, between 125 and 200 cells where counted from at least three independent pituitaries.

### GFRa2+ cells express pituitary specific factors and stem cell markers in murine and human pituitaries

**Figure 2:**
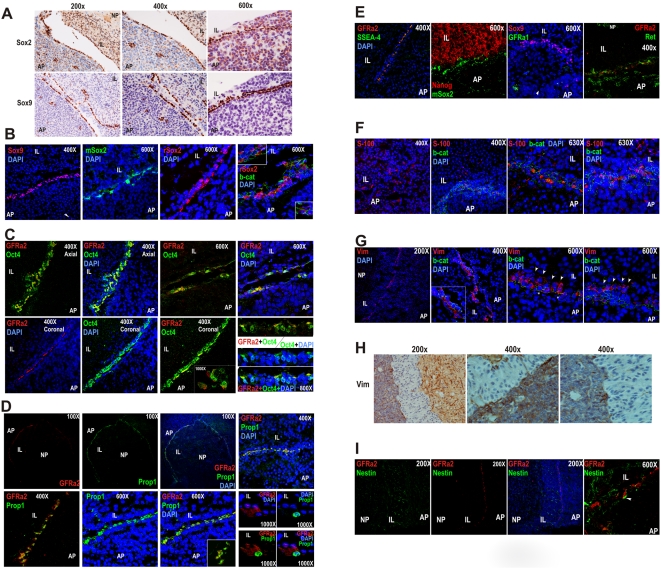
The GPS Niche: GFRa2 cells express Prop1 and stem cell markers while neighbor cells express Vimentin. A) Detection of Sox2 and Sox9 in the mouse and the rat pituitary (mSox2: mouse monoclonal and rSox2: rabbit polyclonal anti-Sox2 antibodies). (B). Sox2 signal co-localizes with b-Catenin. C) In rat pituitary, Oct4 is also expressed in the same line of cells, and co-localizes with GFRa2. D) Co-localization between GFRa2+ and Prop1 in the marginal zone between the AP and IL. Notice the nuclei positive for Prop1 surrounded by the GFRa2 membrane staining. E) GFRa2 cells co-localize with SSEA4, a glycolipid characteristic of Stem cells, but not with Nanog, which is restricted to the IL. GFRa2 cells do not express GFRa1 (which is however observed in somatotrophs) but weakly express the Ret receptor (Fig. S2). F) S-100 is expressed by the folliculostelate cells of the IL and AP of rat pituitary, but is also concentrated in around half of the b-Catenin/GFRa2 cells. G) Vimentin, a mesenchymal stem cell marker, is also expressed in the same niche as the GFRa2 cells but not in the same cells. Towards the IL, a parallel line of elongated cells (arrows) just beyond the b-Catenin/GFRa2 cells (asterisks) can be observed; fixation provokes sometimes the separation of both lines of cells (right panel). A similar Vimentin staining is seen in mouse pituitary (H). I) Although Nestin is expressed in the three portions of the pituitary, GFRa2 cells are negative for Nestin expression. Thin structures similar to axons apparently coming from the Nestin+ neuropituitary contact the GFRa2 cells (arrowhead).

A variety of stem/progenitor cell markers is also expressed in the niche of GFRa2+ cells (Table 1). Among them, the recently described [10] Sox 2 and Sox9 transcription factors (Figure 2A –mouse- and 2B-rat-) that co-stain with GFRa2/b-Catenin. These cells also display a clear signal for Oct4 (Figure 2C).

GFRa2 cells does not express the pituitary specific transcription factor Pit1 (Figure S2-A) but display a clear and specific signal for Prop1 (Figure 2D). Prop1 is a transcription factor known for its exclusive expression in pituitary development. Mutations in the Prop1 gene cause hypopituitarism due to Combined Pituitary Hormone Deficiency (CPHD) in humans [23] and the Ames dwarfism in mice [22], [25].

Based on the fact that GFRa2+ cells express a pituitary specific factor, Prop1, with clear physiological relevance (see Discussion), and *bona-fide* stem cell markers such as Sox and Oct4 proteins, we call them GPS (**G**FRa2+, **P**rop1+, **S**tem). GFRa2+ cells also express SSEA4 (Figure 2E), a glycolipid marker of embryonic stem cells However, the niche of cells is negative for other stem markers such as Nanog –expressed in the IL (Figure 2E) and S2B-, Sox4, Isl-1 or Pax6 (Figure S2 and Table 1). Whereas the other co-receptor, GFRa1, is not expressed in the GPS cells, they are positive for the Ret receptor although with less intensity than somatotrophs (Figure 2E and S2-C).

The calcium-binding protein S-100 (a marker of folliculostellate cells [28]–[30]) is present in about 50% of GPS cells, in addition to many scattered and elongated cells in the AP, MZ and IL (Figure 2F). Another marker of folliculostellate cells, Vimentin [31], [32], delineates the GPS niche (Figure 2G). However, double immunofluorescence with beta-catenin does not show the expected co-localization. Vimentin+ cells appear as a line of elongated cells posterior to the GPSs just before the IL both in rat and mouse pituitaries (Figure 2G, H). Finally, Nestin, a marker of some folliculostellate cells [33] is expressed in long and thin processes through the AP, IL and NP similar to neurons or to folliculostellate cells, but it does not correlate with GFRa2 staining (Figure 2I).

**Figure 3:**
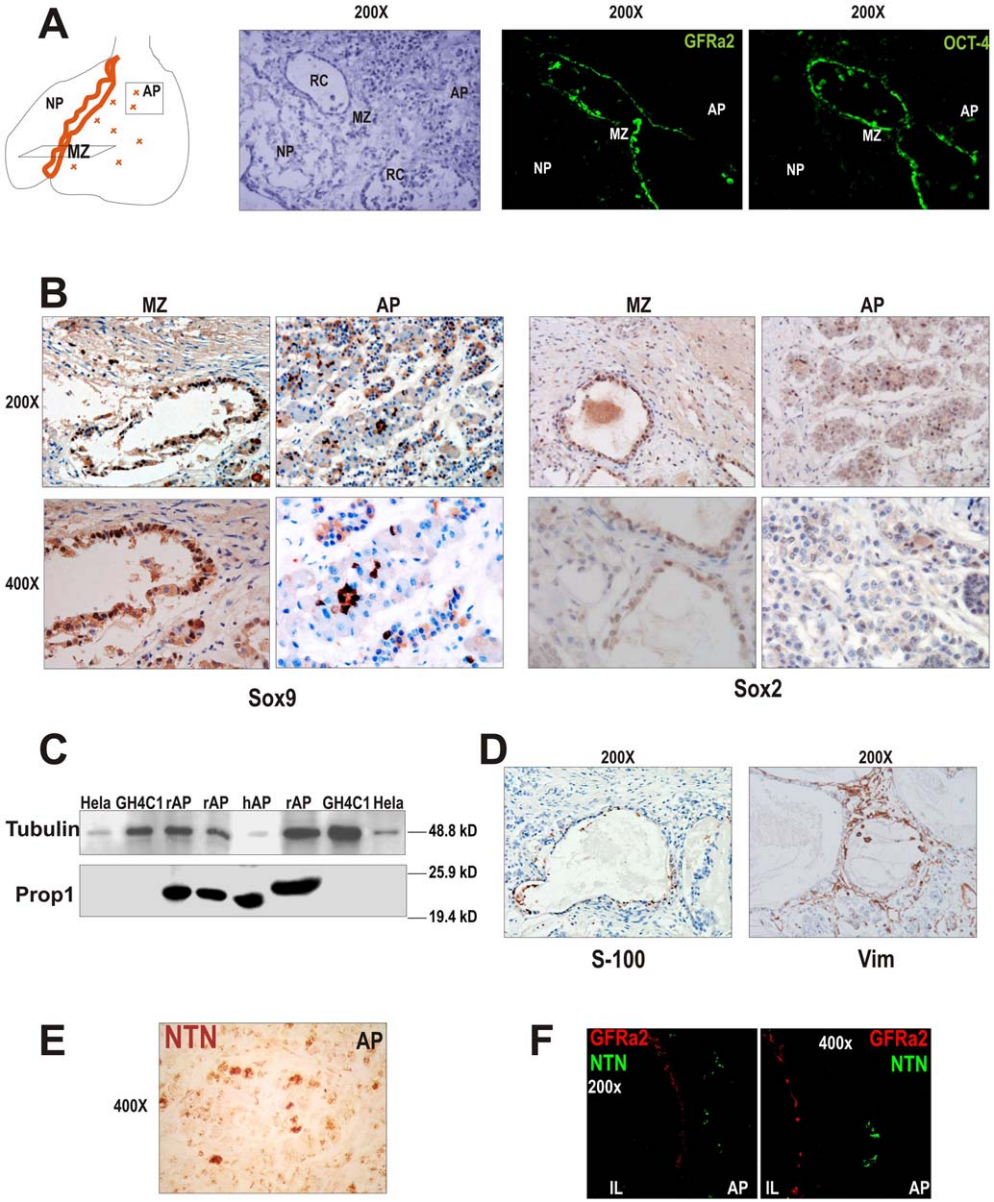
The human marginal zone (MZ) of the pituitary contains a similar niche of GPS cells. A) Cartoon representing the anatomy of the human pituitary with the anterior AP and a posterior NP; the boundary is called MZ and contains dilated structures usually called Rathke's remnant's Cysts (RC). Cells lining the RC express GFRa2 and Oct4. B) These cells also express Sox9 and Sox2. The human pituitary also contains small groups of Sox9+ or Sox2+ cells within the AP. C) Western blot detection of the pituitary specific factor Prop1 protein in rat (rAP) and human (hAP) pituitary, but not expressed in HeLa cells or a somatotroph pituitary cell line (GH4C1). D) S-100 is expressed in around half of the human GFRa2 cells lining the RC, similarly to what observed in the rat pituitary. Similarly, Vimentin+ elongated cells surrounded the GFRa2 epithelium (right panel). E) The GFRa2 ligand NTN is expressed in the human and rat (F) pituitary, and localizes exclusively at the AP.

A similar niche of GPS cells, expressing GFRa2, Oct4, Sox2, Sox9 and is also present in the MZ of the human pituitary around the so called Rathke's remnant cysts (Figure 3A–B). The human pituitary also expresses Prop1 (Figure 3C). The niche of human GPS is also partially positive for S-100 but negative for Vimentin, which is expressed by elongated cells in the same area just in contact with the GPS (Figure 3D). On the other hand, the GFRa2-specific ligand NTN is exclusively expressed in groups throughout the AP and not at the niche either in human or in rat pituitary (Figure 3E–F and Figure S2-F). This finding, together with the planar polarity found in the GPS niche, suggests a functional asymmetric signaling in which the GFRa2/RET/NTN pathway may be implicated.

### GFRa2+ cells form embryonic-like spheroids capable to differentiate in hormone-producing cells

**Figure 4:**
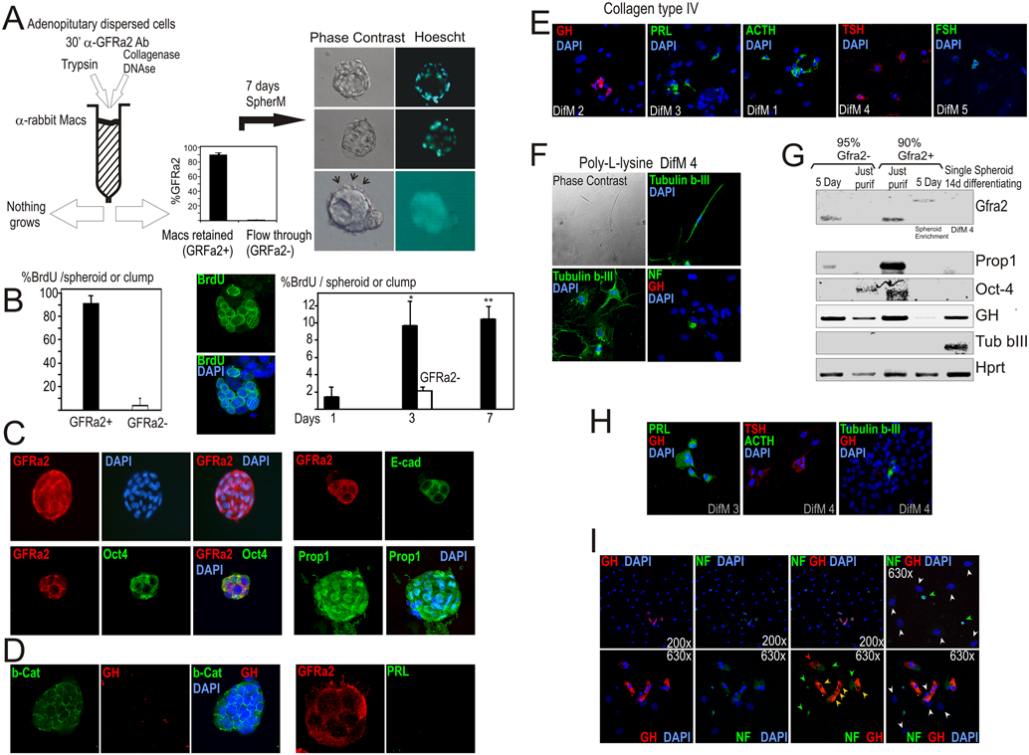
Purified GFRa2+ cells form embryonic-like spheroids that differentiate into different ectodermic cell types. A) Rat AP single-cell dispersions are prepared by treatment with Collagenase. The two fractions obtained, a GFRa2+ purified fraction (90% positivity for GFRa2 by immunofluorescence) and the Flow through GFRa2- (95% negativity for GFRa2), are then kept in SpherM. After 7 days, spheroids formed by small cells are observed in the GFRa2+ fraction. Some of they contain a hollow cavity while others are compact. A bunch of moving cilia is frequently observed in one pole of these spheroids (arrows; see videos in Supplementary Information). AP cell dispersion with trypsin does not result in viable spheroids as GFRa are extracellular receptors sensitive to trypsin treatment. B) Proliferation in the GFRa2+ and GFRa2- fractions after 5 days in the presence of BrdU. Center: A 7-day-old spheroid incubated with BrdU only for the last 12 hours before fixation. Right: BrdU uptake within growing spheroids incubated with BrdU during the last 12 hours before fixation. C) The spheroids are clonal (see Figure S3-A) and express GFRa2, Oct4, Prop1 or E-cadherin. D) These structures express b-Catenin but not hormones such as GH, PRL (and S3-B) or ACTH (data not shown). E–I) A single spheroid was transferred under the microscope to a collagen/poly-lysine-coated well and attached to the matrix with serum for 24 h. Spheroid structure disappear and cells spread through the well. Cells differentiate depending on the culture conditions into E) different pituitary secretory types (intermediate nuclei) or F) neurons (small or big nuclei) showing Tubulin beta III-+ cells+ or NF+ cells. G) mRNA expression of GFRa2/Prop1/Oct4 in the GFRa2+ purified fraction. The GFRa2+ fraction still have some contaminating secretory cells expressing GH. Five days later (spheroids) RNA expression of GFRa2 shifted (alternative splicing) while Prop/Oct4 were negative (even if the proteins were present). 14 days after differentiation of a single hand-picked spheroid, expression of either secretory (GH) or neuronal (Tubulin b-III) differentiation markers is detected. H) Double immunofluorescence in differentiated spheroids showed that differentiation is most frequently driven towards either secretory or neural phenotype. I) However, in some wells double GH/NF+ cells (orange arrows) together with single GH+ (red arrows) or NF+ (green arrows) or negative (white arrows) cells coexisted (Table 2).

To address the differentiation potential of the GPS niche, we isolated GRFa2+ individual cells and maintained them as a suspension culture in a serum-free conditioned medium (SpherM). 2500 cells either GFRa2+ or GFRa2−negative were seeded in a 35-cm-diameter dish (around 800 cells/ml). After seven days, while the GFRa-negative dishes presented a few clumps of cells (12 clumps/dish, 4–8 cells/clump), the GFRa2+ cells formed spheroid structures either compact or hollow with an empty cavity surrounded by small cells (>139 spheroids/dish, around 40 cells/spheroid; average of more than 50 experiments). Some of the spheroids contain cilia and display active movements (Figure 4A and Videos S3, S4, S5 and S6). One of the roles of planar polarity in embryogenesis is to induce oriented cilia during morphogenetic migration, and functional defects in these cilia cause embryonic abnormalities [34]. In humans, GPS are located around the reminiscent Rathke's cleft (see Figure 3), which has been described to present cilia [35], [36]. A benign non-neoplastic disease called Rathke's cleft cyst (RCC) is in fact characterized by a MZ cyst pathognomonically surrounded by ciliated cells [37]. These cysts are thought to originate from remnants of the Rathke's pouch and, in fact, their localization is similar to what shown in Figure 3 for human GPS cells.

The spheroids actively divide up-to 50 cells (Figure 4B). Opposite, the GFRa2 negative fraction maintained in parallel remains as isolate cells with a few 4–8 cell clumps and does not grow (BrdU-negative, Figure 4B, right, white bars). To demonstrate that the spheroids are clonal, i.e. originate from a single cell, we diluted the GFRa2+-cell suspension to 18 cells/ml and seeded 1 ml/well in the first column of a 24-well dish. Dilutions 1∶2 were performed in the next columns up to 0.5 cells/well. After five days spheroids were carefully looked out and all of them photographed to count approximately the number of cells per spheroid (Figure S3-A, a representative experiment with quadruplicates is shown). All the spheroids found were multicellular (ranging from 25 to >100 cells/spheroid). The number of individual spheroids per well were proportional to the number of individual GPS cells seeded per well. Even diluting at 0,5 cell/ml we found near one multicellular spheroid per well.

The spheroids maintain GRFa2 expression and display positive labeling for Oct-4, Prop1, E-cadherin, and b-Catenin but are hormone-negative (Figure 4C–D and Fig S3-B). The GFRa2 ligand, Neurturin (50 ng/ml) has a trophic effect in the spheroid number when culturing in a diluted (0.5×) SpherM (Figure S3-C) indicating the GPS dependence of a functional RET/GFRa2/NTN pathway. If the pituitaries are dispersed with trypsin (instead of Collagenase) no single spheroid grows from the few GFRa2+ purified cells. This may be explained by possible deleterious effects of trypsin in the extracellular domain of GFRa2. Similarly, if the spheroids are dispersed with trypsin, they are able to make secondary spheroids albeit the number of secondary spheroids obtained was 1/3 of the number of GPSs seeded.

In the presence of gelatin and conditioned-media from MEFs (50% MEFM), these GFRa2/Prop+ cells attach to the well and grow slowly as a scattered culture (Figure S3-D). However, after the second passage, differentiated structures as “cord-like” structures, colonies expressing red pigmentation or other kind of defined-cells appear under the microscope intermingled with the scattered GFRa2+ cells. We don't know at present if the GPSs have multipotent capacity. When the GPS cells are cultured on top of mitomycin-treated MEFs in the presence of the characteristic medium for Stem cells (StemM), they grow as undifferentiated colonies and display cilia (Figure S3-C and Video S7). In the presence of MEFM supplemented with LIF (ESGRO), these cells do not attach to the gelatin-coated dish but grow as floating spheres. We have been able to maintain these cultured GPS cells either as attached/floating colonies or spheres at least up to the 7th passage and still continue (Figure S3-E).

We next asked whether GFRa2+ spheroids maintain the capability to differentiate to endocrine cells. Single spheroids were isolated by pipetting under the phase-contrast microscope and placed on Collagen Type IV coated wells, the collagen characteristic of basal membranes from epithelial layers. We next induced attachment with serum for one day, followed by incubation in medium containing a specific combination of supplements (DifM 1–4, see Methods). The spheroid got attached during the first 24 hours of culture in presence of serum. From that moment on, the cells start to attach to the dish and the spheroid progressively disappears. Some of the cells migrate very far away from the point where the spheroid attaches. If the spheroid was big many cells appear on the dish; if the spheroid was small less reduced numbers appear. That means that although we cannot exclude the possibility of proliferation after the induction of attachment/differentiation we have the repeated impression from the many experiments that the differentiated cells do not proliferate. Using this approach, we were able to differentiate these spheroids into GH-, PRL-, TSH-, ACTH-, or FSH-producing cells (Figure 4E). We did not observed any cell positive for GFRa2 or Prop1 after differentiation (Table 2). When spheroids are seeded on top of Poly-L-lysine with DifM4, Tubulin-beta-III positive cells are observed. Tubulin-beta III is characteristic of neurons and in fact some of these cells present bipolar appearance (Figure 4F), suggesting an ectodermal stem cell potential for pituitary GRFa2+ cells. Similar results were obtained using the characteristic neuronal intermediate filament protein Neurofilament (NF).

**Table 2:** Summary of all the differentiation experiments with the 5 differentiation media (DifM1-5)*.

	GH		PRL		bTSH		ACTH		bFSH		Tub b III		NeuroF	
	n°+	nuclei	n°+	nuclei	n°+	nuclei	n°+	nuclei	n°+	nuclei	n°+	nuclei	n°+	nuclei
**DifM1**	n.t.		n.t.		0	10			n.t.		n.t.		n.t.	
							9	38						
							10	18						
**DifM2**	**1(1)**	25							**5 (1)**	25	n.t.		n.t.	
	**1(1)**	52					**12(1)**	52						
			0	17										
			0	17										
					*6 (0)*	29	*0 (0)*	29						
					*7 (0)*	9	*0 (0)*	9						
	*8(0)*	27							*0 (0)*	27				
	**3(2)**	58							**2 (2)**	58				
**DifM3**	*0 (0)*	17	*6 (0)*	17			n.t.		n.t.		n.t.		n.t.	
					0	15								
			6(**a**)	51										
	0 (**b**)	18												
			2(**a**)	3										
	0	13												
**DifM4 (Polylysine)**							n.t.		n.t.		5	15		
					**26(0)**	**27**					**1(0)**	27		
					*7 (0)*	28					*0 (0)*	28		
	**6(5)**	93											**10(5)**	93
	*3 (0)*	9											*0 (0)*	9
	*7 (0)*	37									*0 (0)*	37		
	*0 (0)*	117									*2 (0)*	117		
	*0 (0)*	50											*1 (0)*	50
	*0 (0)*	25									*24 (0)*	25		
			0	18										
**DifM5**	n.t.		n.t.		n.t.		n.t.		2	31	n.t.		n.t.	

* A single isolated spheroid was induced to attach to the gelatine-coated (DifM1,2,3 and 5) or poylysine-coated (DifM4) well with serum for one day, and induced to differentiate during 15 days in the presence of any of the DifM 1 to 5. Wells were fixed and immunofluorescence performed. In some wells double immunofluorescence was performed (aligned in the same row): those wells where more than one cell type co-existed are written in bold and the number of cells double positives for both markers is shown in brackets; those wells where one single type of cell was detected are in italics. In some of the wells (underlined), co-immunofluorescence for GFRa2 (**a**) or Prop (**b**) was performed, always with a negative result.

To evaluate RNA expression throughout the differentiation process, we performed RT-PCR analysis in the GFRa2+ fraction (90% pure), the GFRa2-negative fraction (95% pure), in both fractions 5 days after culture in SpherM (when there is a spheroid-enrichment in the GFRa2+ fraction), and in the cells obtained after differentiation of a single spheroid in DifM4 (Figure 4G). GPS stem markers are strongly expressed in the GFRa2+ fraction and absent in GFRa2-negative cells. In parallel, GH expression is still present after purification, probably due to the abundance of somatotrophs in the pituitary (10% contaminating GFRa2-cells). After culturing in SpherM medium, expression of GPS markers decay although the proteins are still present in the spheroids, and GFRa2 band shifts to a different spliced band. No differentiation markers, Tubulin-beta III, or a very weak band of GH can be detected. It seems that the GPS cells in the spheroid structures start to change its characteristics but need later inputs from different ligands (present in the various DifM) to get differentiated; some of these differences are downregulation of Oct4, Prop1 mRNA expression or alternative splicing of GFRa2 mRNA. In the GFRa2- fraction (95% GFRa2-) with days positivity for GFRa2 and Prop1 mRNA appear demonstrating the self-renewal capacity of the few remaining GFRa2+ cells.

When the spheroids are induced to differentiate, no GPS markers are detected but GH and Tubulin-beta III are expressed de novo. In our hands, differentiation protocols are quite specific since lactotrophs (PRL) are only obtained with DifM 3, whereas GH was never detected in this medium (Table 2). When two markers were simultaneously analyzed in the same well, the majority of differentiated cells are positive for one marker and there was only one type of differentiated cell (blue on Table 2, Figure 4F and H-left). However on occasion two types of differentiated cells co-existed on the same well (green on Table 2, Figure 4I, lower magnification), and even there were some cells positive for two hormones or for GH and NF (pink on Table 2, Figure 4I, higher magnification). In general, positivity for hormones is linked to a nucleus of intermediate size; positivity for NF correlates with a nucleus of small size; and positivity for Tubulin-beta III is frequently observed in cell with a large nucleus.

### Slow proliferation and long telomeres in the GPS niche of adult animals

**Figure 5:**
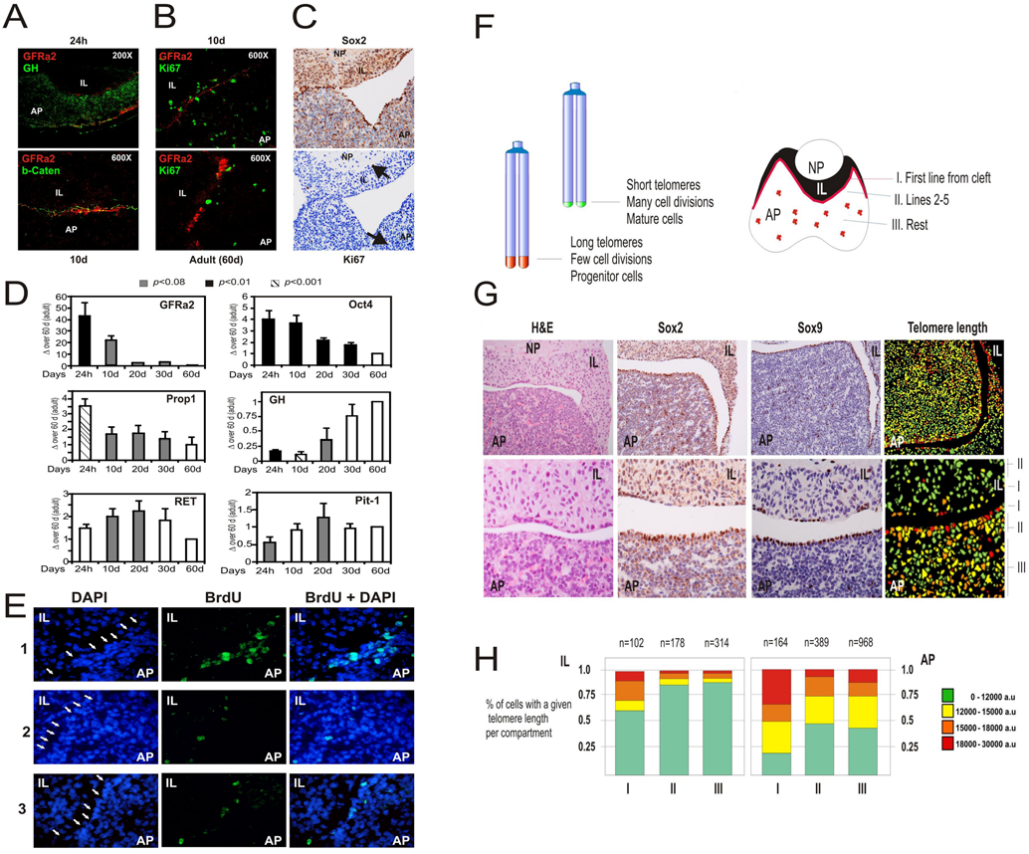
GFRa2+ niche is present at birth and maintained through adulthood with reduced proliferation and long telomeres. A) Newborn (24 h) and 10-days (10 d) old rat pituitaries present a GFRa2+/b-Catenin+ but GH- niche similar to that adult pituitaries (60 to 90-days old). B) 10 d pituitaries display abundant cell proliferation in the GPS niche, as seen by Ki67 staining, opposite to the adult rat (60 d) or mouse organs (C). D) Expression of GFRa2, Oct4, Prop1, GH, Ret and Pit1 in the AP of newborn and 10-, 20-, 30- and 60-day-old rats as detected by qRT-PCR. GPS progenitor markers (GFRa2, Oct4, Prop1) decrease with age while somatotroph markers (Ret, Pit-1) peak around puberty, day 10 to 20, or increase with grow to adulthood (GH). E) BrdU retaining in the GPS niche (arrows). Adult-pituitary nuclei within the niche retain BrdU injected in the rats as newborns. Three different animals (1–3) are depicted in the figure. F) Telomapping analysis of normal mouse pituitaries demonstrates a thin line of very long-telomere containing nuclei exactly in the first row of cells at the IL/AP boundary (regions I) matching the GPS niche. The following rows of cells towards the AP or the IL/NP present a shortening of the telomeres while the bulk of secretory cells have short telomeres characteristic of differentiated cells. G) Normal pituitaries were stained with H&E, Sox2 or Sox9 showing the GPS cells in the AP/IL boundaries (AP region I and IL region I) and some scattered groups through the AP (mostly in region III of the AP). Telomapping analysis as quantified in H) indicates that the region I of AP contains most long-telomere cells. This percentage progressively decreases in region II and III, where scattered GPS cells with long telomeres are found. In the IL, the only cells with long telomeres are also located in region I of the IL. AP, adenopituitary; IL, intermediate lobe; NP, neuropituitary.

In vivo the stem cells are slow cycling cells that retain the nuclear DNA label of infancy into adult age as demonstrated for mouse skin, mammary gland, endometrium and liver [38]–[41], or for rat pancreas and kidney [42], [43]. The GPS niche already exists in newborn-rat pituitaries (Figure 5A). GRFa2+ cells actively divide during early postnatal development but loose proliferative potential with age, as detected by Ki67 staining of the GPS niche (Figure 5A and B). Also in adult mouse pituitaries, cell division is scarce and rarely observed in GPS cells (Figure 5C), thus suggesting that GPSs were slow cycling cells in vivo. In parallel, the expression levels of stem cell markers in the AP decrease during postnatal development to adulthood in an inverse correlation with the production of hormones or the Pit1 transcription factor (Figure 5D), implying a division of stem cells to increase pituitary mass. To analyze the frequency of replication in the putative stem cell niche, we used the BrdU retaining technique. In rats, GRFa2+ cells specifically retained BrdU even 60 days after an injection when newborns whereas this signal was lost in most of the other cells in the pituitary (Figure 5E). These results suggest that the niche of GFRa2 cells replicates slowly after birth, a property shared by most progenitor/stem cells.

Slow replication is linked to long telomeres and these two features are a hallmark of stemness [44]–[46]. Mature cells have usually undergone many divisions and telomere length gradually decreases with each cell cycle due to incomplete replication of telomeric DNA. We have used a novel technique, “telomapping”, to quantify the length of telomeres in situ based upon the specific in-situ hybridization of a fluorescent telomeric DNA probe on paraffin sections. The longest telomeres in the pituitary specifically mark the marginal zone within the IL/AP boundary where GPS cells are located (Figure 5G). Progressive rows of cells towards the AP or NP present less intense signals (orange) while the mature secretory cells in AP have the faintest signal (green) corresponding to short telomeres. These results suggest that progenitor cells in the pituitary are located to the MZ where GPS localize. On the other hand, most cells in the AP display short telomeres suggesting an abundant component of mature cells that have undergone many cell divisions.

### Altered cell cycle regulation of GPS cells in genetically-modified mouse models with hypo- or hyperplastic pituitaries

**Figure 6:**
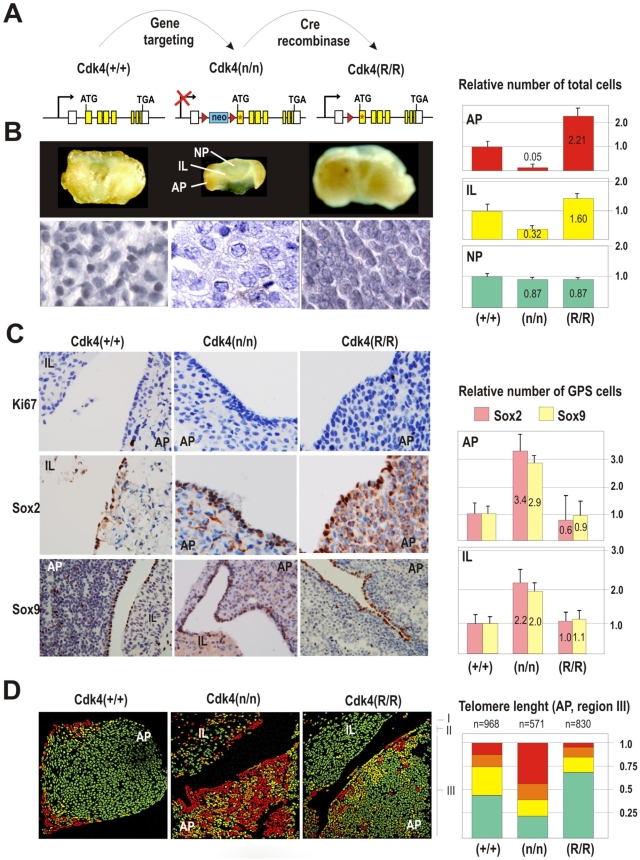
Increased relative abundance of GPS progenitors and decreased formation of endocrine cells in Cdk4–null mice. A) Alleles used in the analysis of GPS cells in a hypopituitarism model. The Cdk4-null allele Cdk4(n) is obtained by insertion of a neo-resistance cassette in Cdk4 intron 1. This mutation is rescued by expressing, through Cre recombination, a Cdk4^R24C^ mutant allele that encodes a hyperactive Cdk4 [48]. B) Cdk4-null mice display a hypoplastic pituitary much smaller than the wild type due to low cellularity and smaller size. These differences mostly affect the AP, containing around 5% cells of the wild-type pituitaries by 2-month age. This phenotype is rescued in the Cdk4(R/R) which displays a bigger pituitary with a 2-fold increase in the AP by 2-months. C) Genetic ablation of Cdk4 does not affect the structure of the stem cell niche. Moreover, the relative abundance of GPS cells is enlarged presenting more cell layers and 3–3.5-fold more GPS cells in the AP and 2-fold in the IL. D) The overall length of telomeres is significantly increased in the Cdk4-deficient AP distal region III. About 48% of these cells display long telomeres whereas this number is about 12% in wild-type or Cdk4^R24C^-rescued AP. AP, adenopituitary; IL, intermediate lobe; NP, neuropituitary.

Proliferation in the progenitor/stem cell niches depends on Cdk4 activity, being carefully downregulated within the niche and increasing when the progenitor cell enters in the so-called transit-amplifying state to become differentiated [47]. Cdk4-deficient mice in which Cdk4 has been inactivated by the insertion of a neomycin-resistant (neo) cassette [Cdk4(n/n) mice] [48] display hypoplastic pituitaries with a dramatic decrease of all hormone-secretory cells in the AP during postnatal life (Figure 6A–B, center and Figure S4). These Cdk4-null pituitaries display normal Ki67 staining during embryonic development but a decreased proliferation after birth (data not shown), similarly to that we have previously observed in Cdk4-null endocrine cells in the pancreas [49]. Yet, the ratio between GPS cells and total number of cells in the pituitary was not only maintained but enlarged in these animals suggesting normal production of these cells during embryonic development but abnormal differentiation into hormone-producing cells. Thus, whereas wild-type mice contain about 0.9% of GPS cells (see Figure S1), these cells display a relative 3-fold enrichment in Cdk4(n/n) mice (Figure 6C). Moreover Cdk4-null pituitaries display long telomeres throughout the AP (Figure 6D), suggesting a reduced number of cell cycles in these small anterior pituitaries. Hypoplastic Cdk4 KO pituitaries do not exhibit an enriched “niche” in absolute terms. The niche is relatively enriched considering the reduced number of endocrine-producing cells. The interpretation is in keeping with the concept that most defects in stem cell proliferation result in a defect in differentiated cells without affecting the stem compartment. A similar situation has been found previously in the hematopoietic compartment of Cdk4/6 deficient embryos [50].

Interestingly, these three phenotypes (reduced pituitary size and cellularity, relative increase in GPS cells and long telomeres in the AP) are rescued when Cdk4 is re-expressed [Cdk4(R/R) mice] by expressing Cre recombinase and removing the neo cassette (Figure 6A–D, right panels), in parallel with the recovery of normal pituitary function (Figure S4). These results suggest that Cdk4 participates in the control of postnatal proliferation and/or differentiation of GPS cells.

## Discussion

The existence of a primordial cell in the pituitary was proposed more than ten years ago when exceptional human pituitary adenomas were observed to concomitantly express Pit1-dependent hormones (GH, PRL and TSH) plus ACTH and gonadotrophic hormones [51]–[53]. More recently, the presence of stem cells in the pituitary has been suggested in dispersed cultures isolated by citometry as a Side Population (SP cells) of mouse pituitary cells positive for Sca1, Nestin, Nanog and Oct4, but negative for Prop1 [7]. Additional progenitor cells have been also proposed as a colony-forming population of Sca1+ and angiotensin-converting enzyme (ACE)-+ cells [9]. Some of the later were in fact located to the MZ of the pituitary. The MZ had been proposed to harbor stem/progenitor cells originated from the Rathke's pouch from which the endocrine cells could be produced [1], [9]. Recently, Sox2+/Sox9− cells have been found in the mouse MZ but also strongly dispersed throughout the pituitary intermingled with secretory cells [10]. A population of Nestin+ cells has been traced after birth in the pituitary in vivo [11]. Nestin+ cells were found in the three parts of the pituitary, and only a small population of the adult secretory AP cells was originated from these Nestin+ cells postpuberally, more than 2.5 months after birth. At present, it is unclear whether the growth of the pituitary after birth or maintenance of the adult population of secretory cells requires a single or several types of progenitor/stem cells.

We have characterized a specific cell population in the MZ of the rodent –rat and mice– and human pituitary, initially identified by the expression of the GFRa2 receptor. These cells exhibit unique features, i.e. not present in other pituitary cell-types, such as the presence of GFRa2 receptors, the expression of the pituitary specific transcription factor Prop1, and the presence of additional stem cell markers such as Sox2, Sox9, SSEA4 and Oct4. The presence of these markers, long telomeres, and the in vitro potential of GPS cells to differentiate in all AP endocrine cells make them strong candidates for the maintenance of differentiated cells on the pituitary. GPS cells are Nanog/Nestin-negative but similar Oct-4+ Multipotent Adult Progenitor Cells (MAPCs) have been reported to be negative for other embryonic stem cell markers such as Nanog or Sca1 [54]. SSEA-4 is an embryonic stem cell marker in humans but it is not present in mouse embryonic stem cell lines, which are instead positive for SSEA-1. There is not known in much detail what markers are present on the surface cells of the early rat embryo stages, if they would be lacto-series of glycolipids (SSEA-1) as in mouse or globo-series of glycolipids (SSEA-3 and SSEA-4) as in humans. Rat embryonic stem cells recently obtained expressed SSEA-4, SSEA-3 and SSEA-1 on top of Oct4 or Nanog [55]. In agreement with these results, it was known that a small percentage of rat Dorsal Root Ganglia (DRG) “small” precursors were positive for SSEA-4 [56]. The DRG is one of the niches where neural crest progenitors/stem cells reside ([57] and many previous references therein). The mouse vs. human difference present into embryonic stem cells, changes in adult stem cells. Recently, it has been demonstrated that hematopoietic stem cells are positive for SSEA-4 (and Sca1 positive but c-Kit/CD45/Flk-1/and SSEA-1 negative) both in mouse and in human and, in fact, SSEA-4 has been proposed to better purify HSCs from the bone marrow [58].

Adult stem cells divide infrequently and reside in protected microenvironments or niches [59] with a low rate of telomere erosion throughout their life-time. These niches can be either acellular or contain other cell types that give support to the stem cell niche [60]. The presence of Vimentin+ cells near the GPS cells in both rodent and human pituitary suggests the presence of a cellular stem cell niche on the pituitary. GPS cells also express E-cadherin and b-Catenin in a polarized manner (coronal vs. axial) surrounded by Vimentin+ cells, suggesting a putative relation with the known function of the Wnt/b-Catenin pathway together with E-cadherin to retain the stem cells within the niche confines [60]. Future studies assessing a role of the canonical Wnt pathway on this niche are clearly merited. Perpendicular staining of GFRa2 versus b-Catenin within the GPS niche reminds of planar polarity, a specific coordination of an epithelial layer of cells to behave with a physiological direction (recently reviewed in [27], [61]. It is therefore not casual that isolated GPS cells in culture form moving embryonic-like spheroids and present specialized cilia. One of the roles of planar polarity in embryogenesis is indeed to induce oriented cilia during morphogenetic migration to prevent embryonic abnormalities [34]. The planar polarity in the GPS niche also suggests a functional asymmetric signaling in which both the GFRa2 and Wnt pathways may be implicated. Thus, the Ret/GFRa2 pathway may help to indicate the cells in the niche the correct side to migrate and/or to proliferate. The expression of the ligand NTN in discrete cells through the AP but not in the niche cells adds an interesting suggestion of luring the GPS cells out of the niche through a guiding gradient. This situation is reminiscent of the niche present in the seminiferous tubules of the testes where Ret and GFRa1/GFRa2, along their ligands GDNF and NTN, play an important role in the interplay between multipotency versus differentiation of the germ stem cells [18], [19], [62]. Similarly, in the pituitary, the NTN/GFRa2 axis may modulate stem/progenitor physiology whereas Ret/GFRa1 system controls somatotroph differentiation and fate (death versus survival) through Pit1 regulation as we have described previously [63].

Some additional genes expressed by the GPS niche, such as Sox proteins or Prop1, have important roles in pituitary physiology and disease. Mutations in the Sox2 gene cause pituitary hypoplasia associated with hypogonadotrophic hypogonadism and eye, ear and encephalic abnormalities [64]–[67]. Ames dwarf mice and Prop1 null mice have a normal pituitary volume at birth but the organ does not grow nor differentiate in postnatal life. Similar phenotype have the patients affected by Combined Pituitary Hormone Deficiency (CPHD) [23], a hypopituitarism caused by mutations in Prop1 [68] where there pituitary undergo progressive hormone loss suggesting a depletion of progenitors. Patients with CPHD display a general loss in all types of secretory cells, but affecting more those that are required throughout growth and puberty (GH, LH/FSH) and metabolism (TSH). Although initially it may be not present, delayed ACTH deficiency also appears [69]. Some patients present with hyperplasia of the pituitary while others display hypoplastic pituitaries; moreover, it is currently accepted that enlargement of the pituitary precedes the hypoplasia [69], [70]. Ames dwarf mice have a slightly different phenotype, with the predominant failure in the Pit1-dependent secretory types (GH, PRL, TSH) and apparently not deficiency in gonadotrophs/corticotrophs [22], [71]. Since this in a spontaneous mutation we cannot be sure of the genetic background. However, Prop1-deficient mice have display a phenotype similar to the human CPHD, including gonadotroph deficiency [25]. Both Ames dwarf and Prop1-deficient mice have a normal (or only slightly decreased) pituitary at birth, suggesting a defect in adult homeostasis. Moreover, Prop1 transgenic mice have a delay in puberty [72]. Prop1 in the pituitary embryonic progenitor cells of the Rathke's pouch is considered to play a role in the migration process of the progenitor cells out of the marginal zone [26], [73]. Our data demonstrate that Prop1 expression in the adult pituitary is restricted to the GPS niche. It is tempting to speculate a role for Prop1 in protecting the stem cells and correctly guide them through asymmetric division/differentiation when needed. A detailed study of the niche in these animal models will be performed.

Interestingly, the pituitary deficiency induced by Prop1 mutations is reminiscent of the hypopituitarism induced by inactivation of the cell cycle regulator Cdk4 ([74] and Figures 6 and S4). GPS cells are present in this model although accompanied by an overall decrease in endocrine producing cells. These AP cells display longer telomeres suggesting a defective number of cell divisions from their progenitor cells. Interestingly, GFRa2-deficient mice display a significant failure to thrive after weaning although the involvement of pituitary function in this phenotype has not been addressed [16]. Also similarly to Cdk4, persistent Prop1 expression in the mouse delays endocrine differentiation and enhances tumor susceptibility [72] (see below).

The initial Rathke's pouch, as well as the encephalic neural tube, comes from the anterior ectoderm. GFRa2+ spheroids are able to differentiate to secretory pituitary cells but also towards neuron-like phenotypes when driven appropriately with a specific differentiation medium. A similar induction of neuronal phenotypes from epithelial stem cells of the inner ear has been demonstrated ([75]. Similarly, pituitary secretory cells can be obtained from neuronal fetal progenitors [76] and many human pituitary adenomas present with neural metaplasia [77]. However, the GPS are able to remain undifferentiated when grown in conditioned-medium from MEFs; in this conditions, however, part of the cells differentiate spontaneously with passages. GPS remain undifferentiated and form colonies when grown directly on top of MEFs or when grown in the presence of ESGRO (LIF), a feature shared by all stem cells described.

All together, our results suggest that GPS cells may have relevant contributions to postnatal pituitary homeostasis. These cells a likely to form a functional niche of adult precursor cells with functional relevance in the physiological expansion of the pituitary gland throughout life as well as protection from pituitary disease.

## Materials and Methods

For a detailed list of methods and antibodies and dilutions see Supplementary Methods S1 and Table S1, S2 and S3.

### Human and murine samples

Rats were obtained from the Central Animal House of the USC, a registered animal facility that maintains the animals under welfare and ethical conditions complying with the 86/609/CEE, RD223/88, and OM 13/10/89 laws. The project had the approval of the Ethical Committee of the USC. Rat pituitaries were obtained from adult (200–250 gr., 60 days) male/female Sprague-Dowley rats. To study expression during postnatal development newborns, 10, 20, 30 and 60 days old male rat pituitaries were compared.

Human pituitary samples were selected from the archives of the Department of Pathology, Hospital Universitario Virgen del Rocío (Sevilla, Spain). Informed consent was required from patients according to the policies of the Ethical Committee of the Hospital.

Generation and characteristics of the Cdk4-deficient mice has been previously described [48], [78]–[80]. The Cre strain used was CMV-Cre [48]. Mice were maintained in a mixed 129/Sv×C57BL/6J background following the institutional guidelines at the Spanish National Cancer Research Center (CNIO) and the protocol approved by the Committee of Bioethics and Animal Care of the Comunidad de Madrid. The animals were observed in a daily basis and sick mice were euthanized humanely in accordance with the Guidelines for Humane End Points for Animals used in biomedical research.

### Immunodetection

For immunofluorescence, rat pituitaries were oriented and immersed in an OCT-filled plastic cryomold (Sakura) and frozen inside a glass beaker filled with isopentane previously immersed in liquid N2; frozen cryomolds were maintained at −80°C until sectioned in 10 microns cryosections. The sections were fixed with 0.1% Paraformaldehyde for 10 minutes (GFRa2, Prop-1, Oct-4, Citokeratins, E-cadherin, SSEA-4, rabbit Sox2, Nanog, Nestin, ACTH, PRL, FSH, LH) or with -20°C methanol for 5 minutes (GFRa2, Prop-1, Oct-4, b-Catenin, rabbit anti-Sox2 (rSox2), Nanog, Ret, GFRa1, GH, TSH) or 0.5% paraformaldehyde for 20 minutes (GFRa2, GH, Ki-67); for mouse anti-Sox2 (mSox2) and Sox9 the pituitaries were fixed in 4% paraformaldehyde overnight before freezing and sectioning. Alternatively, cryosections were fixed in 4% paraformaldehyde for at least 10 minutes. Primary antibodies were applied overnight in PBS, thoroughly washed in PBS followed by 1 hour incubation with secondary antibodies, washing and mounted using GelMount (Biomeda). Guinea pig polyclonal antibody anti- Prop1 was made in house against the carboxy-terminal domain of mouse Prop-1. cDNA encoding amino acids 151 to 223 were cloned downstream of either a GST or a His-tag vector. Fusion proteins were expressed in BL21 E. coli and partially purified over glutathione agarose (Sigma) or Ni2+-NTA-agarose (Qiagen). Initial immunizations were performed with GST-Prop-1 fusion protein and the final boosts were performed with the His-Prop-1 fusion proteins. It has been already demonstrated that this antibody recognizes Prop1 transcription factor in mouse E12.5 [81]. Double immunofluorescences were performed in consecutive days; to prevent secondary antibody backgrounds, the order was dependent on the species of the primary antibody: first day goat, guinea pig or rabbit, second day rabbit or mouse respectively. Negative (using PBS instead of primary antibody) and preadsorption (competing with cold peptide/protein) controls were routinely run in parallel (see Supplementary Methods S1). Nuclei were counterstained with 20 µg/ml DAPI (Sigma). A TCS-SP2-DMRE Confocal Microscope with Ar, He/Ne 543 and He/Ne 633 Lasers (Leica) and LCS software was used to analyze the results.

For immunocytochemistry and telomapping, mouse or human pituitaries were fixed in 10% buffered formalin at 4°C, dehydrated through graded alcohols and xylene, and embedded in paraffin. Prior to embedding, pituitaries were oriented in order to obtain specific sagittal or coronal 5 microM sections. Prior to IHC, paraffin-embedded slides were de-paraffinized, re-hydrated, immersed in 10 mM citrate solution and epitopes retrieved by three high-power, 5 min microwave pulses. Slides were washed in water, blocked in 1∶10 dilution of normal goat serum (Vector Labs) and incubated with primary antibodies. Slides were then incubated with secondary biotinylated antibodies followed by signal development with an immunoperoxidase reagent (ABC-HRP, Vector Labs) and DAB (Sigma). Sections were lightly counterstained with hematoxylin and analyzed by light microscopy.

For immunoblotting, tissues o cells were lysed as previously described [63], [82].

### Isolation and culture of GFRa2+ cells

A detailed protocol is provided as Supplementary Information. Briefly, freshly isolated cell suspensions were prepared from male rat or mouse pituitaries using magnetic activated cell sorting (MACS; Miltenyi) or a fluorescence-activated cell sorter (FACS; FACSAria, Becton-Dickinson). The experiments with spheroids shown in the Figures 4 and S3 were performed with rat cells, although many have been reproduced in mouse cells (data not shown).

GFRa2+ purified cells were cultured in un-coated wells in the presence of SpherM. After 5–7 days spheroids were either video-recorded or fixed for further immunofluorescence or induced to differentiate. For the BrdU-uptake experiments, 10 microM BrdU (Sigma) was added from the beginning, but a toxic effect was seen with longer treatments than 5 days; to evaluate the % of cell division in spheroids of different days BrdU was added for the last 12 hours of incubation before fixation. To differentiate each spheroid was carefully picked with a P1000 pipet under the microscope and placed in poly-L-lysine or Collagen type IV treated Cultureslides (BD) in 10% FCS-SpherM. The following day, the medium was replaced during 14 days by any of the differentiated media DifM 1–4. Immunofluorescence of spheroids was performed pipetting them on top of 8 microM inserts (Millipore) and fixing them with 70% Ethanol at room temperature during 30 minutes, plus 4 M HCL during 20 minutes (BrdU labeling) or with −20°C Methanol for 5 minutes for the other antibodies before proceeding as above. Differentiated cells were fixed in Methanol (hormones) or in 4% paraformaldehyde for 20 minutes (hormones, Tubulin-beta III, NF).

### BrdU retaining technique

Three days old rats were injected subcutaneously with 50 µg/g BrdU (Sigma) in 0.9% NaCl twice/day during 3.5 days. 60 days later, animals were sacrificed and pituitaries frozen as above. Cryosections were fixed in −20°C Methanol for 10 minutes, washed and incubated in 4 M HCl for 20 minutes. After washing, immunofluorescence with anti-BrdU (BD) was performed as above.

### Confocal quantitative telomere FISH (Telomapping)

For telomapping, paraffin-embedded tissue sections were hybridized with a PNA-tel Cy3-labelled probe and telomere length was determined as described [46]. DAPI, Cy3 signals were acquired simultaneously into separate channels using a confocal ultraspectral microscope (Leica TCS-SP2-A-OBS-UV) using a PL APO 20×/0.70 PH 2 as lens with Leica LCS software and maximum projections from image stacks (10 sections at steps 1.0 microM) were generated for image quantification. The DAPI image was used to define the nuclear area and the Cy3 image to quantify of telomere fluorescence. The binary DAPI mask was applied to the matching Cy3 to obtain a combined image with telomere fluorescence information for each nucleus. Cy3 fluorescence intensity (telomere fluorescence) was measured as “average gray values” (total gray value/nuclei area) units (arbitrary units of fluorescence). These “average telomere fluorescence” values always represent the average Cy3 pixel intensity for the total nuclear area, and not the average value of individual telomere spot intensities, therefore ruling out that differences in nuclear size may influence telomere length measurements.

## Supporting Information

Figure S1 Expression of GFRa2 and b-Catenin in the adenopituitary A) GFRa2 mRNA expression in the rat adenopituitary (AP) is comparable with the testes, a gland well known for its GFR alpha expression. B) GFRa2 stains about 0.9% of all AP cells detected by flow cytometry after specific binding of anti-GFRa2 antibody. The enzyme dispersed suspension of mouse-adenopituitary cells were sequentially incubated with anti-GFRa2 antibody followed by FITC-anti-rabbit antibody (see Supplementary Tables S1 and S2). The cell suspension was analyzed by cytometry; in red the analyzed FITC+ population and in blue the sorted population presenting the strongest FITC signal. In the negative control (only Ig, secondary antibody) this population was less than <0.1%, while in the GFRa2+ samples accounted for around 0.9% of the total cell suspension. The GFRa2+ population is composed of homogeneous small cells as seen by the low level of the sorted population on the FSC in comparison with the non-FITC population or with the faint FITC+ within the control. C) Low magnifications of a whole section of a rat pituitary (DAPI) and the b-Catenin enrichment at the niche between AP and IL. The only pituitaries small enough to picture like this were from 10-days old rats. AP, adenopituitary; IL, intermediate lobe; NP, neuropituitary.
(0.92 MB PDF)

Figure S2 The GPS niche is weakly positive for RET but does not express Pit-1, Nanog, GFRa1, Nestin or Sox4. A) The GPS niche is negative for Pit1, a pituitary transcription factor expressed by somatotrophs (GH), lactotrophs and thyrotrophs, as is negative for GH. B) The GPS niche is also negative for Nanog. Nanog staining is only observed in the IL and does not overlap with b-Catenin at the niche. C) The Ret tyrosine-kinase receptor stains specific cells in the AP (mostly somatotrophs, [1], [2], and it is also expressed in neurons of the NP. It also weakly stains the GFRa2+ niche; however the GPS cells are negative for GFRa1. D) The Nestin+cells of the pituitary are dispersed through the IL and the AP [3], but do not coincide with the GPS. E) Sox4 is expressed in the mouse AP but it is not a marker of the GPS niche. F) Western blot of GFRa2 and Neurturin (NTN) in rat and human adenopituitary. Hela cells are a human positive control for GFRa2. PRL has a slight interspecies difference in MW.
(1.71 MB PDF)

Figure S3 Differentiation and proliferation properties of GFRa2-purified cells in vitro. A) The spheroids are clonal: A representative experiment is shown where GFRa2+ cells were diluted in SpherM to 18 cells/ml and seeded into the first column of a 24-well dish. Further dilutions 1∶2 were performed in the following wells. Five days later all the spheroids per well were counted (white numbers in the middle of the wells) and photographed to be able to appreciate an approximate number of cells/spheroid. In those wells where more than 4 spheroids were found, a picture of four of them is shown. B) GFRa2 spheroids express Prop1 and thin lines of b-Catenin and are negative for PRL. C) Neurturin (NTN), the GFRa2 ligand, functions as a physiological promoter of spheroid formation when cells are cultured under sub-optimal conditions (0.5×: medium diluted by half) of SpherM culture media. D) Three ways of culturing MACS purified GFRa2+ cells render different phenotypes: a) In uncoated dishes with SpherM, GFRa2+ grow as spheroids as described; b) cultured on gelatin-coated dishes using 50% of conditioned medium from MEFs (MEFM), they attach to the surface and grow as GFRa2+/Prop1+ scattered cells. However, with passages some differentiated groups of cells forming cord-like structures or red-pigmented colonies appear and the scattered GPS cell number is less; c) when cultured directly on top of mitomycin-treated MEFs (as frequently used for embryonic stem cells), GFRa2+ cells form colonies that present cilia in the apical pole (Supplementary Video 6). E) Adding Esgro to the MEFM (MEFM+E), the cells did not attach to the gelatin-coated surface, but grew slowly but steadily as compact spheres. They were passaged every 25 days. We show here four independent cultures five days after passage. As expected, GPS cells cultured on top of MEF carried on with passages forming colonies (black arrows), although some isolated differentiated cells appeared.
(1.32 MB PDF)

Figure S4 Cdk4 null mouse but not Cdk4(R/R) has hypopituitarism A) Sagittal microphotographs of pituitaries from Cdk4(+/+), Cdk4(n/n) and Cdk4(R/R) 2-month-old mice. B) The total number of hormone-producing cells is decreased in young (2–4 months-old) Cdk4-deficient mouse pituitaries and they have smaller pituitaries (panel A and Figure 6). However, the relative percentage of hormone-producing cells is not grossly altered in Cdk4-deficient mice, suggesting an overall deficiency in the production of all these cells from these progenitors. C) Adult female mice present a physiological increase in lactotroph cells in comparison with males that is maintained in the Cdk4-null mice, in spite of having a much less number of total lactotrophs. Cdk4(R/R) mice recover normal amount of lactotrophs. In the left, serum prolactin levels in the animals were analyzed by immunoassay. D) Representative images of hormone-producing cells in Cdk4(n/n) mice.
(0.34 MB PDF)

Video S1 Three dimensional reconstruction of the rat AP niche using b-Catenin staining (green).
(1.81 MB AVI)

Video S2 Three dimensional reconstruction of the rat AP niche using all channels together, DAPI (Nuclei) blue, GFRa2 (red membrane staining), and b-Catenin (green).
(1.78 MB MPG)

Video S3 Spheroid with beating cilia in one pole.
(1.05 MB AVI)

Video S4 Hollow spheroid moving against another.
(5.24 MB AVI)

Video S5 Hollow spheroid moving fast through the culture dish.
(3.55 MB AVI)

Video S6 Compact spheroid with cilia.
(2.32 MB AVI)

Video S7 Colony of GFRa2+ cells grown on top of mitomycin-treated MEFs for two weeks, with cilia beating on the surface.
(2.74 MB AVI)

Methods S1 (8.22 MB DOC)

Table S1 List of Antibodies and dilutions. Primary Antibodies
(0.06 MB DOC)

Table S2 List of secondary antibodies and related reagents.
(0.04 MB DOC)

Table S3 Oligonucleotides used to analyze gene expression by RT-PCR.
(0.03 MB DOC)

## References

[1] Vankelecom H (2007). Non-hormonal cell types in the pituitary candidating for stem cell.. Semin Cell Dev Biol.

[2] Allaerts W, Vankelecom H (2005). History and perspectives of pituitary folliculo-stellate cell research.. Eur J Endocrinol.

[3] Horvath E, Kovacs K (2002). Folliculo-stellate cells of the human pituitary: A type of adult stem cell?. Ultrastruct Pathol.

[4] Zhu X, Gleiberman AS, Rosenfeld MG (2007). Molecular physiology of pituitary development: Signaling and transcriptional networks.. Physiol Rev.

[5] Vankelecom H (2007). Stem cells in the postnatal pituitary?. Neuroendocrinology.

[6] Melmed S (2003). Mechanisms for pituitary tumorigenesis: The plastic pituitary.. J Clin Invest.

[7] Chen J, Hersmus N, Van Duppen V, Caesens P, Denef C (2005). The adult pituitary contains a cell population displaying stem/progenitor cell and early embryonic characteristics.. Endocrinology.

[8] Lepore DA, Roeszler K, Wagner J, Ross SA, Bauer K (2005). Identification and enrichment of colony-forming cells from the adult murine pituitary.. Exp Cell Res.

[9] Lepore DA, Jokubaitis VJ, Simmons PJ, Roeszler KN, Rossi R (2006). A role for angiotensin-converting enzyme in the characterization, enrichment, and proliferation potential of adult murine pituitary colony-forming cells.. Stem Cells.

[10] Fauquier T, Rizzoti K, Dattani M, Lovell-Badge R, Robinson IC (2008). SOX2-expressing progenitor cells generate all of the major cell types in the adult mouse pituitary gland.. Proc Natl Acad Sci U S A.

[11] Gleiberman AS, Michurina T, Encinas JM, Roig JL, Krasnov P (2008). Genetic approaches identify adult pituitary stem cells.. Proc Natl Acad Sci U S A.

[12] Arighi E, Borrello MG, Sariola H (2005). RET tyrosine kinase signaling in development and cancer.. Cytokine Growth Factor Rev.

[13] Bespalov MM, Saarma M (2007). GDNF family receptor complexes are emerging drug targets.. Trends Pharmacol Sci.

[14] Airaksinen MS, Saarma M (2002). The GDNF family: Signalling, biological functions and therapeutic value.. Nat Rev Neurosci.

[15] Heuckeroth RO, Enomoto H, Grider JR, Golden JP, Hanke JA (1999). Gene targeting reveals a critical role for neurturin in the development and maintenance of enteric, sensory, and parasympathetic neurons.. Neuron.

[16] Rossi J, Luukko K, Poteryaev D, Laurikainen A, Sun YF (1999). Retarded growth and deficits in the enteric and parasympathetic nervous system in mice lacking GFR alpha2, a functional neurturin receptor.. Neuron.

[17] Meng X, Lindahl M, Hyvonen ME, Parvinen M, de Rooij DG (2000). Regulation of cell fate decision of undifferentiated spermatogonia by GDNF.. Science.

[18] Widenfalk J, Parvinen M, Lindqvist E, Olson L (2000). Neurturin, RET, GFRalpha-1 and GFRalpha-2, but not GFRalpha-3, mRNA are expressed in mice gonads.. Cell Tissue Res.

[19] Hofmann MC, Braydich-Stolle L, Dym M (2005). Isolation of male germ-line stem cells; influence of GDNF.. Dev Biol.

[20] Urbano AG, Suarez-Penaranda JM, Dieguez C, Alvarez CV (2000). GDNF and RET-gene expression in anterior pituitary-cell types.. Endocrinology.

[21] Japon MA, Urbano AG, Saez C, Segura DI, Cerro AL (2002). Glial-derived neurotropic factor and RET gene expression in normal human anterior pituitary cell types and in pituitary tumors.. J Clin Endocrinol Metab.

[22] Sornson MW, Wu W, Dasen JS, Flynn SE, Norman DJ (1996). Pituitary lineage determination by the prophet of pit-1 homeodomain factor defective in ames dwarfism.. Nature.

[23] Wu W, Cogan JD, Pfaffle RW, Dasen JS, Frisch H (1998). Mutations in PROP1 cause familial combined pituitary hormone deficiency.. Nat Genet.

[24] Fofanova O, Takamura N, Kinoshita E, Parks JS, Brown MR (1998). Compound heterozygous deletion of the PROP-1 gene in children with combined pituitary hormone deficiency.. J Clin Endocrinol Metab.

[25] Nasonkin IO, Ward RD, Raetzman LT, Seasholtz AF, Saunders TL (2004). Pituitary hypoplasia and respiratory distress syndrome in Prop1 knockout mice.. Hum Mol Genet.

[26] Ward RD, Raetzman LT, Suh H, Stone BM, Nasonkin IO (2005). Role of PROP1 in pituitary gland growth.. Mol Endocrinol.

[27] Zallen JA (2007). Planar polarity and tissue morphogenesis.. Cell.

[28] Nakajima T, Yamaguchi H, Takahashi K (1980). S100 protein in folliculostellate cells of the rat pituitary anterior lobe.. Brain Res.

[29] Marin F, Boya J, Lopez-Carbonell A, Borregon A (1989). Immunohistochemical localization of intermediate filament and S-100 proteins in several non-endocrine cells of the human pituitary gland.. Arch Histol Cytol.

[30] Itakura E, Odaira K, Yokoyama K, Osuna M, Hara T (2007). Generation of transgenic rats expressing green fluorescent protein in S-100beta-producing pituitary folliculo-stellate cells and brain astrocytes.. Endocrinology.

[31] Marin F, Boya J, Lopez-Carbonell A (1989). Immunocytochemical localization of vimentin in stellate cells (folliculo-stellate cells) of the rat, cat and rabbit pituitary pars distalis.. Anat Embryol (Berl).

[32] Kasper M, Karsten U (1988). Coexpression of cytokeratin and vimentin in rathke's cysts of the human pituitary gland.. Cell Tissue Res.

[33] Krylyshkina O, Chen J, Mebis L, Denef C, Vankelecom H (2005). Nestin-immunoreactive cells in rat pituitary are neither hormonal nor typical folliculo-stellate cells.. Endocrinology.

[34] Park TJ, Haigo SL, Wallingford JB (2006). Ciliogenesis defects in embryos lacking inturned or fuzzy function are associated with failure of planar cell polarity and hedgehog signaling.. Nat Genet.

[35] Shanklin WM (1951). The incidence and distribution of cilia in the human pituitary with a description of microfollicular cysts derived from rathke's cleft.. Acta Anat (Basel).

[36] Yoshimura F, Soji T, Kiguchi Y (1977). Relationship between the follicular cells and marginal layer cells of the anterior pituitary.. Endocrinol Jpn.

[37] Islam O, Butt T, Abrahams J (2007). Rathke cleftt cysts.. Http://wwwEmedicinecom/radio/topic594Htm.

[38] Taylor G, Lehrer MS, Jensen PJ, Sun TT, Lavker RM (2000). Involvement of follicular stem cells in forming not only the follicle but also the epidermis.. Cell.

[39] Welm BE, Tepera SB, Venezia T, Graubert TA, Rosen JM (2002). Sca-1(pos) cells in the mouse mammary gland represent an enriched progenitor cell population.. Dev Biol.

[40] Chan RW, Gargett CE (2006). Identification of label-retaining cells in mouse endometrium.. Stem Cells.

[41] Kuwahara R, Kofman AV, Landis CS, Swenson ES, Barendswaard E (2008). The hepatic stem cell niche: Identification by label-retaining cell assay.. Hepatology.

[42] Duvillie B, Attali M, Aiello V, Quemeneur E, Scharfmann R (2003). Label-retaining cells in the rat pancreas: Location and differentiation potential in vitro.. Diabetes.

[43] Oliver JA, Maarouf O, Cheema FH, Martens TP, Al-Awqati Q (2004). The renal papilla is a niche for adult kidney stem cells.. J Clin Invest.

[44] Moore KA, Lemischka IR (2006). Stem cells and their niches.. Science.

[45] Fuchs E, Tumbar T, Guasch G (2004). Socializing with the neighbors: Stem cells and their niche.. Cell.

[46] Flores I, Canela A, Vera E, Tejera A, Cotsarelis G (2008). The longest telomeres: A general signature of adult stem cell compartments.. Genes Dev.

[47] Horsley V, Aliprantis AO, Polak L, Glimcher LH, Fuchs E (2008). NFATc1 balances quiescence and proliferation of skin stem cells.. Cell.

[48] Rane SG, Dubus P, Mettus RV, Galbreath EJ, Boden G (1999). Loss of Cdk4 expression causes insulin-deficient diabetes and Cdk4 activation results in beta-islet cell hyperplasia.. Nat Genet.

[49] Martin J, Hunt SL, Dubus P, Sotillo R, Nehme-Pelluard F (2003). Genetic rescue of Cdk4 null mice restores pancreatic beta-cell proliferation but not homeostatic cell number.. Oncogene.

[50] Malumbres M, Sotillo R, Santamaria D, Galan J, Cerezo A (2004). Mammalian cells cycle without the D-type cyclin-dependent kinases Cdk4 and Cdk6.. Cell.

[51] Desai B, Burrin JM, Nott CA, Geddes JF, Lamb EJ (1995). Glycoprotein hormone alpha-subunit production and plurihormonality in human corticotroph tumours–an in vitro and immunohistochemical study.. Eur J Endocrinol.

[52] Matsuno A, Sasaki T, Mochizuki T, Fujimaki T, Sanno N (1996). A case of pituitary somatotroph adenoma with concomitant secretion of growth hormone, prolactin, and adrenocorticotropic hormone–an adenoma derived from primordial stem cell, studied by immunohistochemistry, in situ hybridization, and cell culture.. Acta Neurochir (Wien).

[53] Kovacs K, Horvath E, Stefaneanu L, Bilbao J, Singer W (1998). Pituitary adenoma producing growth hormone and adrenocorticotropin: A histological, immunocytochemical, electron microscopic, and in situ hybridization study. case report.. J Neurosurg.

[54] Ulloa-Montoya F, Kidder BL, Pauwelyn KA, Chase LG, Luttun A (2007). Comparative transcriptome analysis of embryonic and adult stem cells with extended and limited differentiation capacity.. Genome Biol.

[55] Ueda S, Kawamata M, Teratani T, Shimizu T, Tamai Y (2008). Establishment of rat embryonic stem cells and making of chimera rats.. PLoS ONE.

[56] Dodd J, Solter D, Jessell TM (1984). Monoclonal antibodies against carbohydrate differentiation antigens identify subsets of primary sensory neurones.. Nature.

[57] Li HY, Say EH, Zhou XF (2007). Isolation and characterization of neural crest progenitors from adult dorsal root ganglia.. Stem Cells.

[58] Gang EJ, Bosnakovski D, Figueiredo CA, Visser JW, Perlingeiro RC (2007). SSEA-4 identifies mesenchymal stem cells from bone marrow.. Blood.

[59] Scadden DT (2006). The stem-cell niche as an entity of action.. Nature.

[60] Xie T, Li L (2007). Stem cells and their niche: An inseparable relationship.. Development.

[61] Lawrence PA, Struhl G, Casal J (2007). Planar cell polarity: One or two pathways?. Nat Rev Genet.

[62] He Z, Jiang J, Hofmann MC, Dym M (2007). Gfra1 silencing in mouse spermatogonial stem cells results in their differentiation via the inactivation of RET tyrosine kinase.. Biol Reprod.

[63] Canibano C, Rodriguez NL, Saez C, Tovar S, Garcia-Lavandeira M (2007). The dependence receptor ret induces apoptosis in somatotrophs through a pit-1/p53 pathway, preventing tumor growth.. EMBO J.

[64] Fantes J, Ragge NK, Lynch SA, McGill NI, Collin JR (2003). Mutations in SOX2 cause anophthalmia.. Nat Genet.

[65] Ferri AL, Cavallaro M, Braida D, Di Cristofano A, Canta A (2004). Sox2 deficiency causes neurodegeneration and impaired neurogenesis in the adult mouse brain.. Development.

[66] Hagstrom SA, Pauer GJ, Reid J, Simpson E, Crowe S (2005). SOX2 mutation causes anophthalmia, hearing loss, and brain anomalies.. Am J Med Genet A.

[67] Kelberman D, Rizzoti K, Avilion A, Bitner-Glindzicz M, Cianfarani S (2006). Mutations within Sox2/SOX2 are associated with abnormalities in the hypothalamo-pituitary-gonadal axis in mice and humans.. J Clin Invest.

[68] Ward RD, Stone BM, Raetzman LT, Camper SA (2006). Cell proliferation and vascularization in mouse models of pituitary hormone deficiency.. Mol Endocrinol.

[69] Bottner A, Keller E, Kratzsch J, Stobbe H, Weigel JF (2004). PROP1 mutations cause progressive deterioration of anterior pituitary function including adrenal insufficiency: A longitudinal analysis.. J Clin Endocrinol Metab.

[70] Vallette-Kasic S, Barlier A, Teinturier C, Diaz A, Manavela M (2001). PROP1 gene screening in patients with multiple pituitary hormone deficiency reveals two sites of hypermutability and a high incidence of corticotroph deficiency.. J Clin Endocrinol Metab.

[71] Andersen B, Pearse RV, Jenne K, Sornson M, Lin SC (1995). The ames dwarf gene is required for pit-1 gene activation.. Dev Biol.

[72] Cushman LJ, Watkins-Chow DE, Brinkmeier ML, Raetzman LT, Radak AL (2001). Persistent Prop1 expression delays gonadotrope differentiation and enhances pituitary tumor susceptibility.. Hum Mol Genet.

[73] Vesper AH, Raetzman LT, Camper SA (2006). Role of prophet of Pit1 (PROP1) in gonadotrope differentiation and puberty.. Endocrinology.

[74] Melmed S (2003). Mechanisms for pituitary tumorigenesis: The plastic pituitary.. J Clin Invest.

[75] Li H, Liu H, Heller S (2003). Pluripotent stem cells from the adult mouse inner ear.. Nat Med.

[76] U HS, Alilain W, Saljooque F (2002). Fetal brain progenitor cells transdifferentiate to fates outside the nervous system.. Mol Endocrinol.

[77] Johnson MD, Fan X, Bourne P, Walters D (2007). Neuronal differentiation and expression of neural epitopes in pituitary adenomas.. J Histochem Cytochem.

[78] Franklin DS, Godfrey VL, Lee H, Kovalev GI, Schoonhoven R (1998). CDK inhibitors p18(INK4c) and p27(Kip1) mediate two separate pathways to collaboratively suppress pituitary tumorigenesis.. Genes Dev.

[79] Latres E, Malumbres M, Sotillo R, Martin J, Ortega S (2000). Limited overlapping roles of P15(INK4b) and P18(INK4c) cell cycle inhibitors in proliferation and tumorigenesis.. EMBO J.

[80] Kiyokawa H, Kineman RD, Manova-Todorova KO, Soares VC, Hoffman ES (1996). Enhanced growth of mice lacking the cyclin-dependent kinase inhibitor function of p27(Kip1).. Cell.

[81] Suh H, Gage PJ, Drouin J, Camper SA (2002). Pitx2 is required at multiple stages of pituitary organogenesis: Pituitary primordium formation and cell specification.. Development.

[82] Garcia A, Alvarez CV, Smith RG, Dieguez C (2001). Regulation of pit-1 expression by ghrelin and GHRP-6 through the GH secretagogue receptor.. Mol Endocrinol.

