# Supplementary figures and images for: A GRFa2/Prop1/Stem (GPS) Cell Niche in the Pituitary
Source: PLoS One. 2009 Mar 13;4(3):e4815. doi: 10.1371/journal.pone.0004815 (PMC2654029; doi:10.1371/journal.pone.0004815)

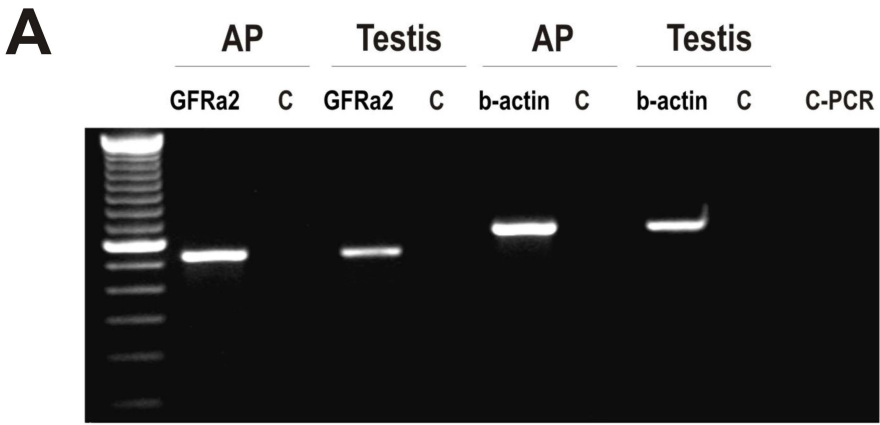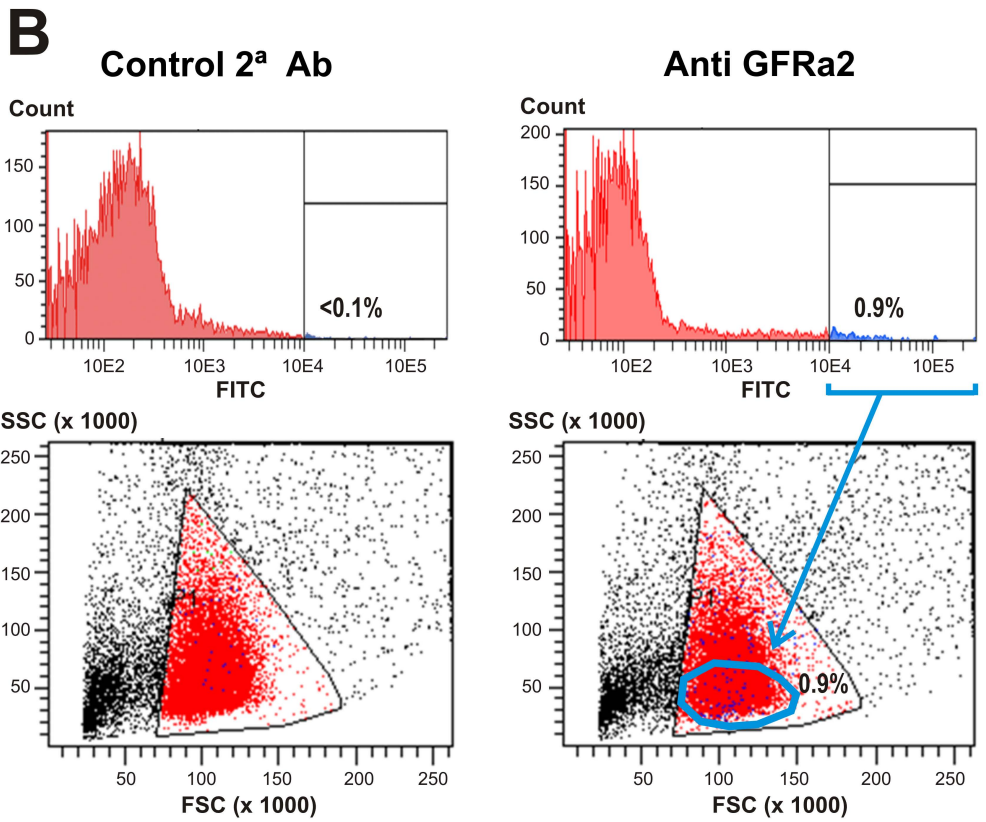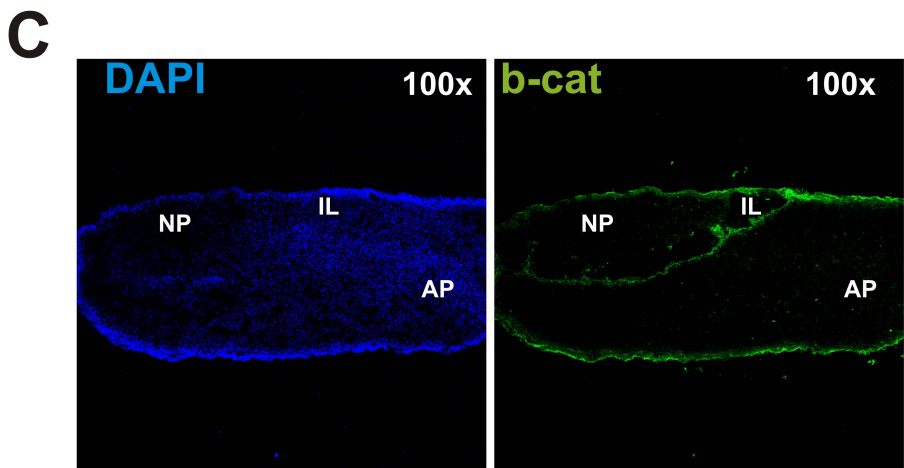

Pituitary 10 days

Supplementary Fig 1

Supplement: Figure S1 — Expression of GFRa2 and b-Catenin in the adenopituitary A) GFRa2 mRNA expression in the rat adenopituitary (AP) is comparable with the testes, a gland well known for its GFR alpha expression. B) GFRa2 stains about 0.9% of all AP cells detected by flow cytometry after specific binding of anti-GFRa2 antibody. The enzyme dispersed suspension of mouse-adenopituitary cells were sequentially incubated with anti-GFRa2 antibody followed by FITC-anti-rabbit antibody (see Supplementary Tables S1 and S2). The cell suspension was analyzed by cytometry; in red the analyzed FITC+ population and in blue the sorted population presenting the strongest FITC signal. In the negative control (only Ig, secondary antibody) this population was less than <0.1%, while in the GFRa2+ samples accounted for around 0.9% of the total cell suspension. The GFRa2+ population is composed of homogeneous small cells as seen by the low level of the sorted population on the FSC in comparison with the non-FITC population or with the faint FITC+ within the control. C) Low magnifications of a whole section of a rat pituitary (DAPI) and the b-Catenin enrichment at the niche between AP and IL. The only pituitaries small enough to picture like this were from 10-days old rats. AP, adenopituitary; IL, intermediate lobe; NP, neuropituitary. (0.92 MB PDF) [file pone.0004815.s001.pdf]

**A**

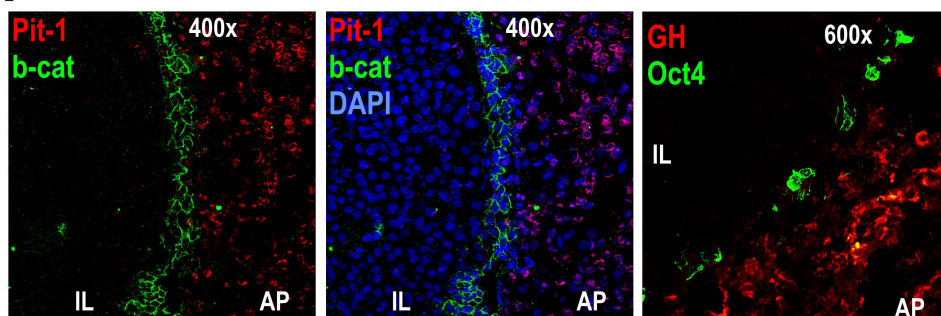

**B**

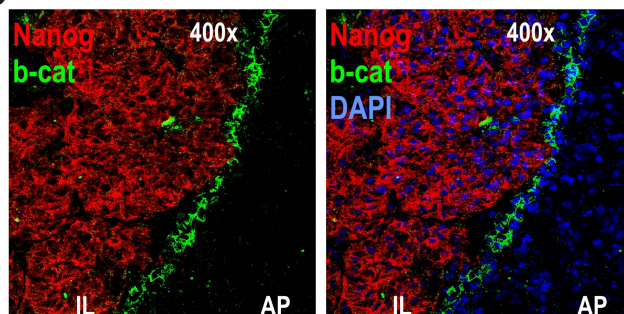

**C**

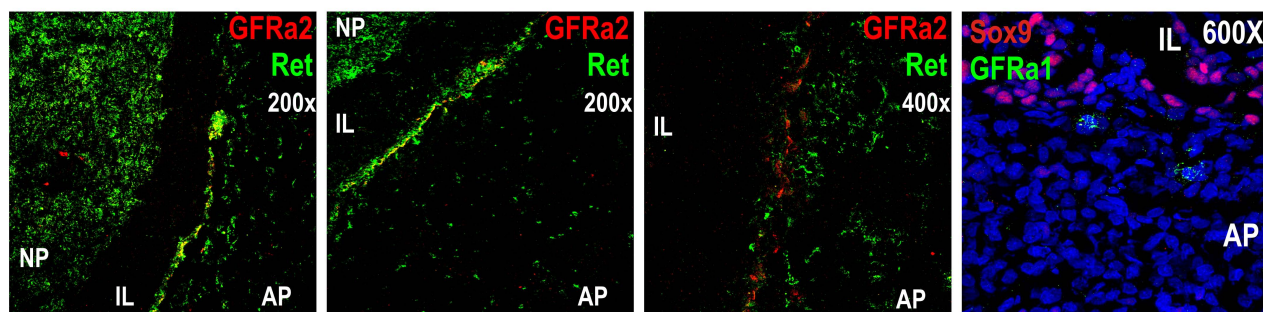

**D**

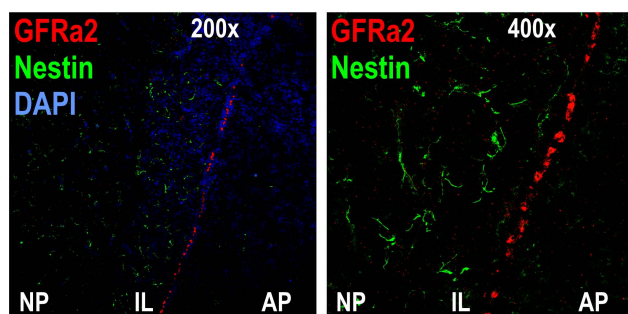

**F**

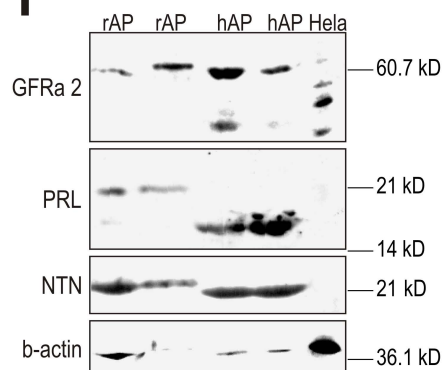

**E**

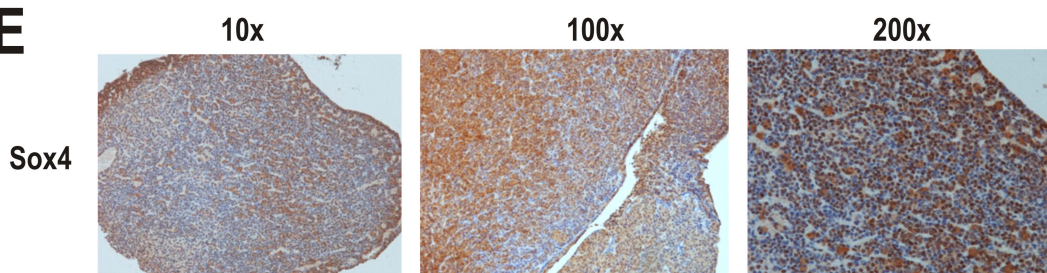

Supplementary Figure 2

Supplement: Figure S2 — The GPS niche is weakly positive for RET but does not express Pit-1, Nanog, GFRa1, Nestin or Sox4. A) The GPS niche is negative for Pit1, a pituitary transcription factor expressed by somatotrophs (GH), lactotrophs and thyrotrophs, as is negative for GH. B) The GPS niche is also negative for Nanog. Nanog staining is only observed in the IL and does not overlap with b-Catenin at the niche. C) The Ret tyrosine-kinase receptor stains specific cells in the AP (mostly somatotrophs, [1], [2], and it is also expressed in neurons of the NP. It also weakly stains the GFRa2+ niche; however the GPS cells are negative for GFRa1. D) The Nestin+cells of the pituitary are dispersed through the IL and the AP [3], but do not coincide with the GPS. E) Sox4 is expressed in the mouse AP but it is not a marker of the GPS niche. F) Western blot of GFRa2 and Neurturin (NTN) in rat and human adenopituitary. Hela cells are a human positive control for GFRa2. PRL has a slight interspecies difference in MW. (1.71 MB PDF) [file pone.0004815.s002.pdf]

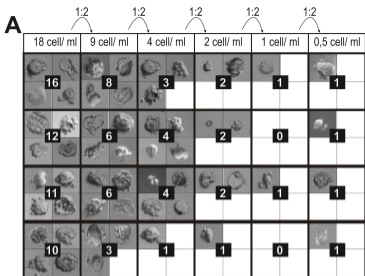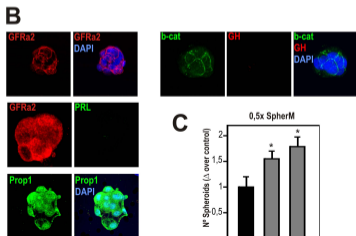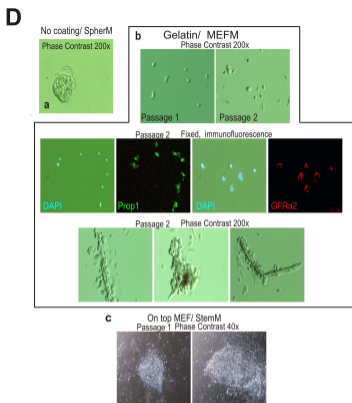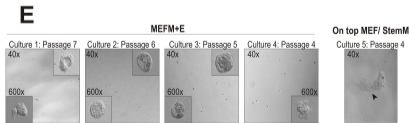

Supplementary Figure 3

Supplement: Figure S3 — Differentiation and proliferation properties of GFRa2-purified cells in vitro. A) The spheroids are clonal: A representative experiment is shown where GFRa2+ cells were diluted in SpherM to 18 cells/ml and seeded into the first column of a 24-well dish. Further dilutions 1∶2 were performed in the following wells. Five days later all the spheroids per well were counted (white numbers in the middle of the wells) and photographed to be able to appreciate an approximate number of cells/spheroid. In those wells where more than 4 spheroids were found, a picture of four of them is shown. B) GFRa2 spheroids express Prop1 and thin lines of b-Catenin and are negative for PRL. C) Neurturin (NTN), the GFRa2 ligand, functions as a physiological promoter of spheroid formation when cells are cultured under sub-optimal conditions (0.5×: medium diluted by half) of SpherM culture media. D) Three ways of culturing MACS purified GFRa2+ cells render different phenotypes: a) In uncoated dishes with SpherM, GFRa2+ grow as spheroids as described; b) cultured on gelatin-coated dishes using 50% of conditioned medium from MEFs (MEFM), they attach to the surface and grow as GFRa2+/Prop1+ scattered cells. However, with passages some differentiated groups of cells forming cord-like structures or red-pigmented colonies appear and the scattered GPS cell number is less; c) when cultured directly on top of mitomycin-treated MEFs (as frequently used for embryonic stem cells), GFRa2+ cells form colonies that present cilia in the apical pole (Supplementary Video 6). E) Adding Esgro to the MEFM (MEFM+E), the cells did not attach to the gelatin-coated surface, but grew slowly but steadily as compact spheres. They were passaged every 25 days. We show here four independent cultures five days after passage. As expected, GPS cells cultured on top of MEF carried on with passages forming colonies (black arrows), although some isolated differentiated cells appeared. (1.32 MB PDF) [file pone.0004815.s003.pdf]

**A**

Cdk4(+/+)

Cdk4(-/-)

Cdk4(R/R)

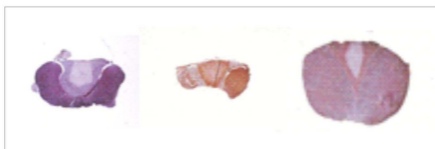**B**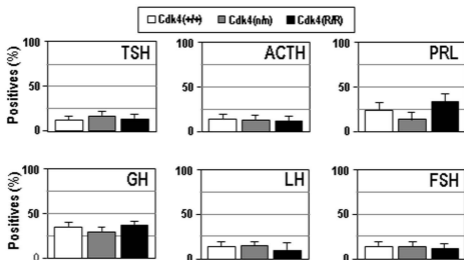**C**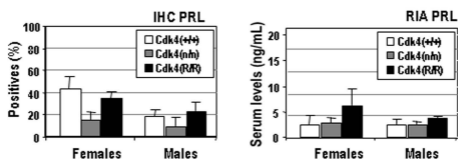**D**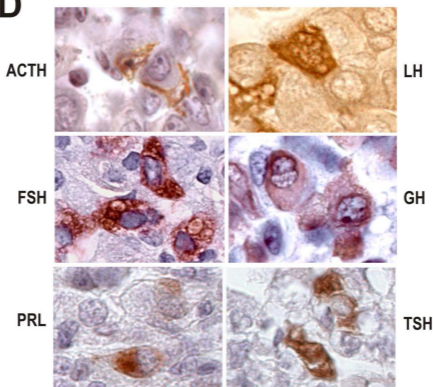

Supplementary Figure 4

Supplement: Figure S4 — Cdk4 null mouse but not Cdk4(R/R) has hypopituitarism A) Sagittal microphotographs of pituitaries from Cdk4(+/+), Cdk4(n/n) and Cdk4(R/R) 2-month-old mice. B) The total number of hormone-producing cells is decreased in young (2–4 months-old) Cdk4-deficient mouse pituitaries and they have smaller pituitaries (panel A and Figure 6). However, the relative percentage of hormone-producing cells is not grossly altered in Cdk4-deficient mice, suggesting an overall deficiency in the production of all these cells from these progenitors. C) Adult female mice present a physiological increase in lactotroph cells in comparison with males that is maintained in the Cdk4-null mice, in spite of having a much less number of total lactotrophs. Cdk4(R/R) mice recover normal amount of lactotrophs. In the left, serum prolactin levels in the animals were analyzed by immunoassay. D) Representative images of hormone-producing cells in Cdk4(n/n) mice. (0.34 MB PDF) [file pone.0004815.s004.pdf]
