# Supplementary material for: A GRFa2/Prop1/Stem (GPS) Cell Niche in the Pituitary
Source: PLoS One. 2009 Mar 13;4(3):e4815. doi: 10.1371/journal.pone.0004815 (PMC2654029; doi:10.1371/journal.pone.0004815)
Supplement: Methods S1 — (8.22 MB DOC) [file pone.0004815.s012.doc]

**A GRFa2/Prop1/Stem (GPS) Cell Niche in the Pituitary**

by Montse Garcia-Lavandeira *et al.*

**Supplementary Methods**

**Immunodetection.** For immunofluorescence, rat pituitaries were oriented and immersed in an OCT-filled plastic cryomold (Sakura) and frozen inside a glass beaker filled with isopentane previously immersed in liquid N2; frozen cryomolds were maintained at -80ºC until sectioned in 10 microM cryosections. The sections were fixed and processed as described in the main text.


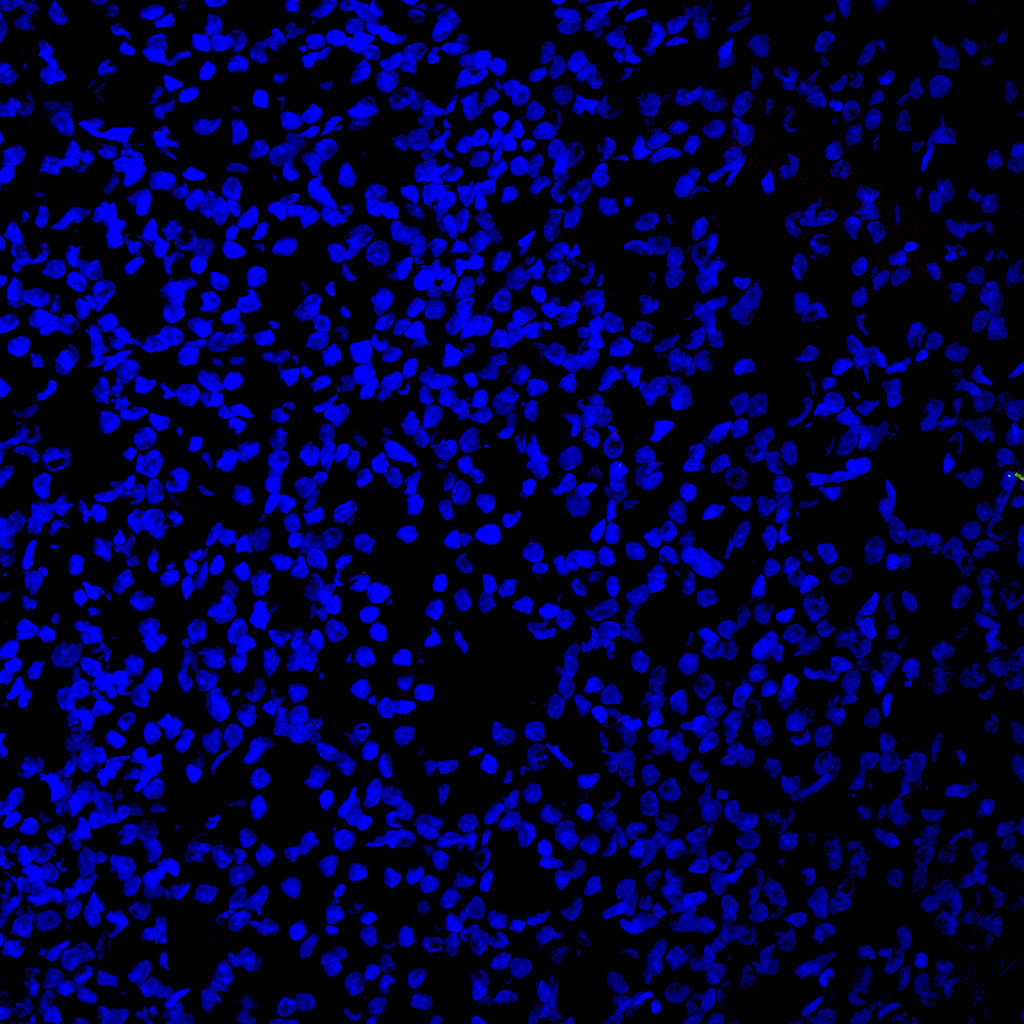
Double immunofluorescences were performed in consecutive days; to prevent secondary antibody backgrounds, the order was dependent on the species of the primary antibody: first day goat, guinea pig or rabbit, second day rabbit or mouse respectively. Negative controls (using PBS instead of primary antibody) were routinely run in parallel (see left picture). Preadsorbed controls, where the antibodies were previously incubated overnight with an excess of the peptide (GFRa2, Abcam), the fusion protein (GST-Prop_155-223_) or the full-length protein (Nanog, Abcam) were also performed; the intensity of the specific signal was practically abolished (see pictures). Nuclei were counterstained with 20 μg/ml DAPI (Sigma). A TCS-SP2-DMRE Confocal Microscope with Ar, He/Ne 543 and He/Ne 633 Lasers (Leica) and LCS software was used to analyze the results.

Immunofluorescence in absence of primary antibodies, using anti-rabbit followed by anti-mouse and counterstained with DAPI (200x)


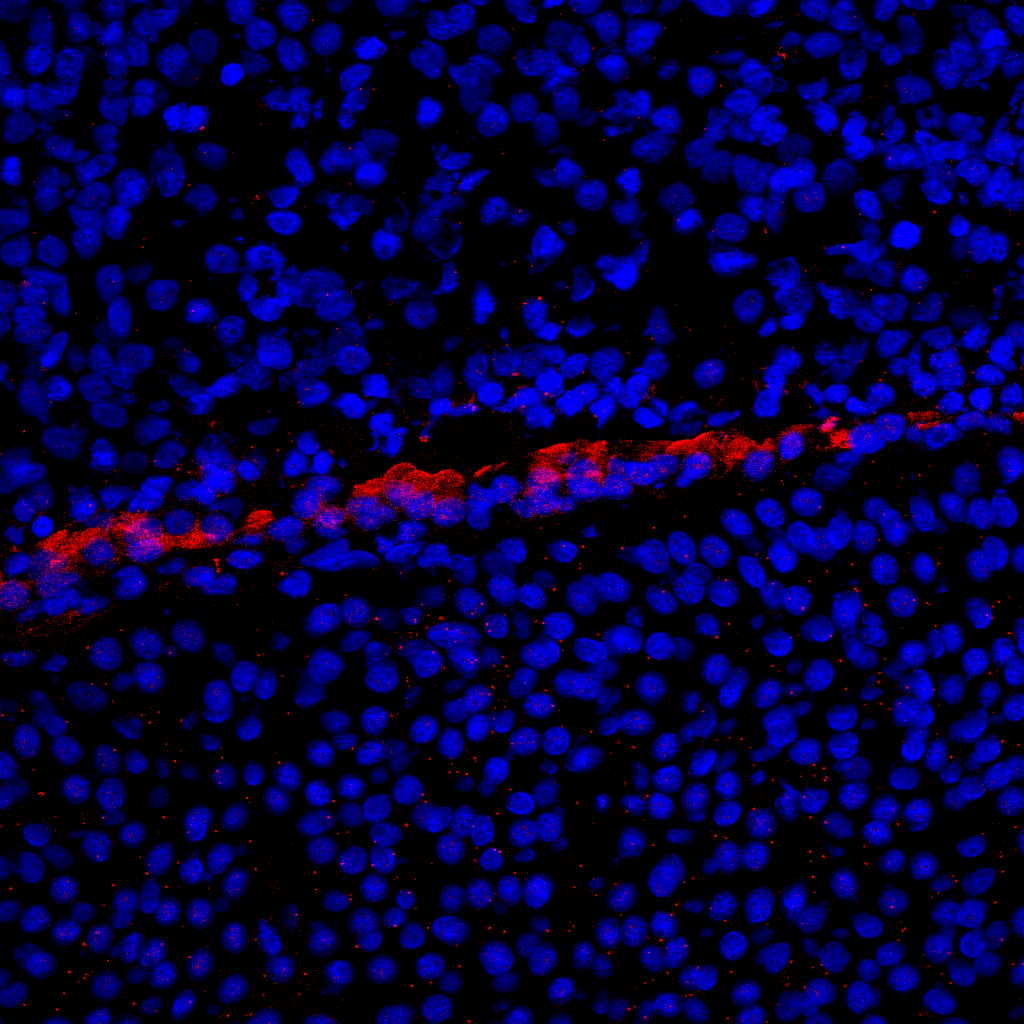

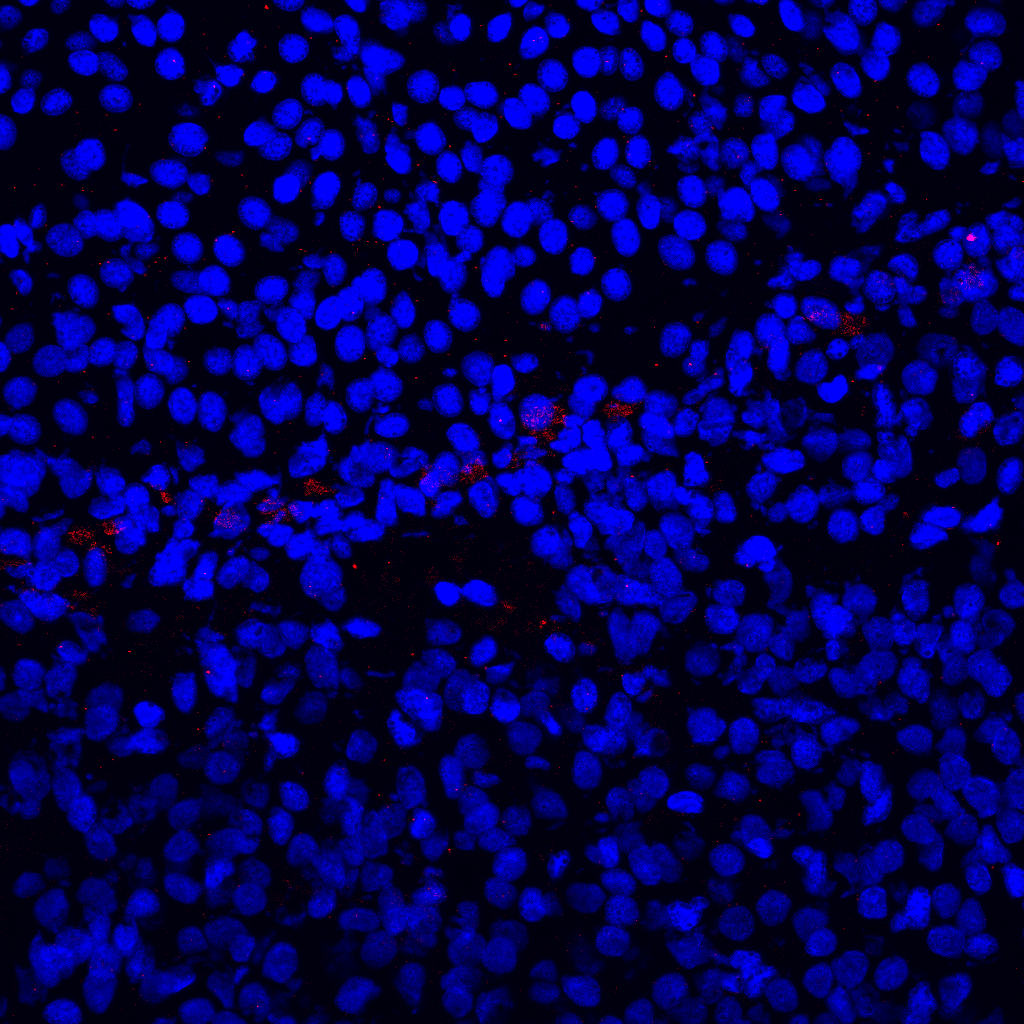

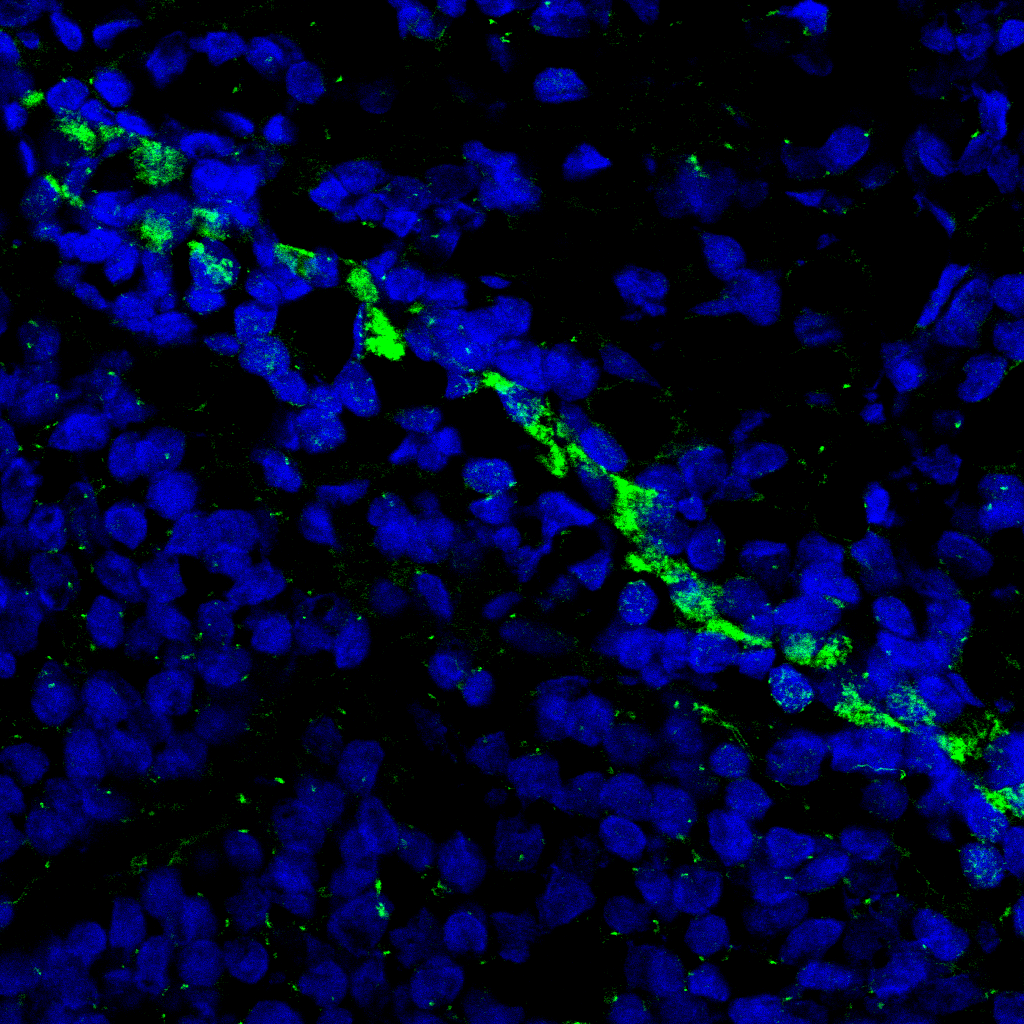

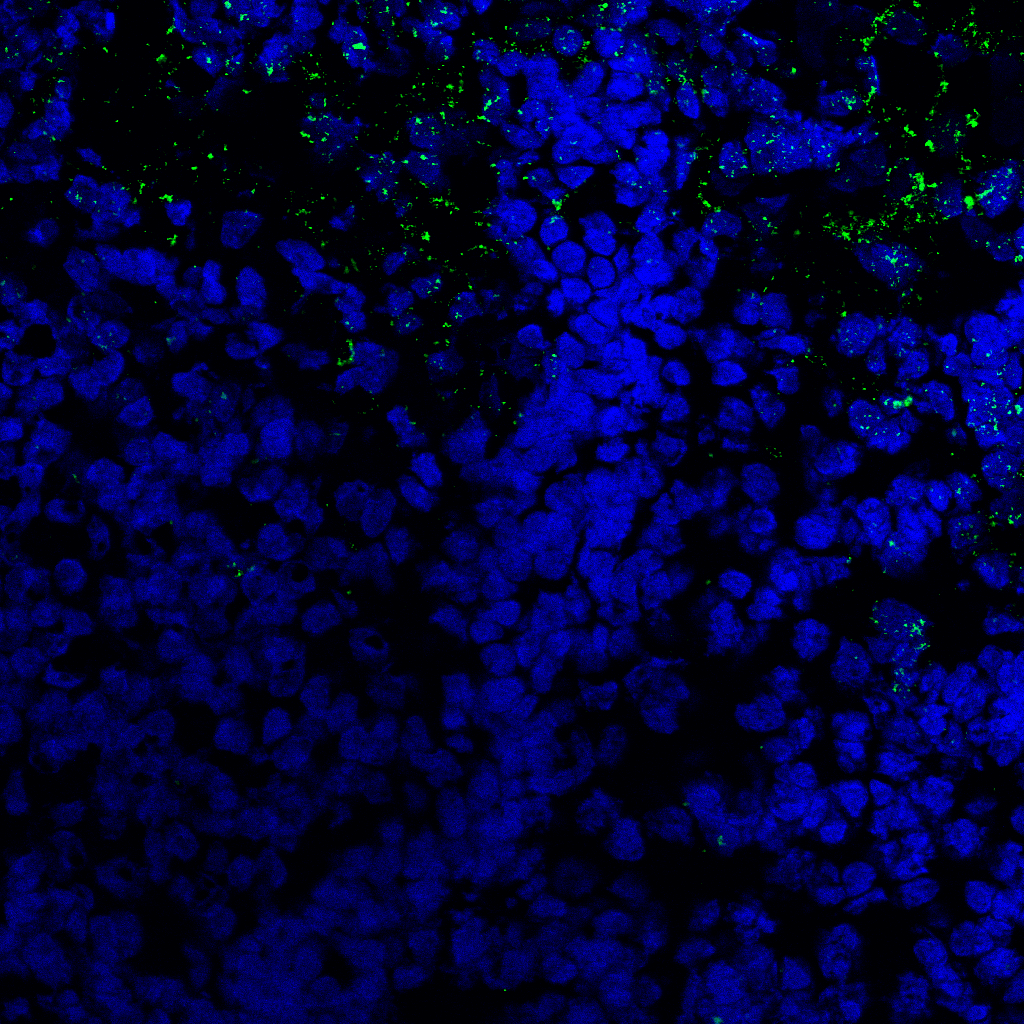


Immunofluorescence

anti-prop1 1:200

Immunofluorescence

anti-prop1 1:200+GST-Prop

Immunofluorescence anti-GFRa2 1:600+GFRa2 peptide

Immunofluorescence anti-GFRa2 1:600


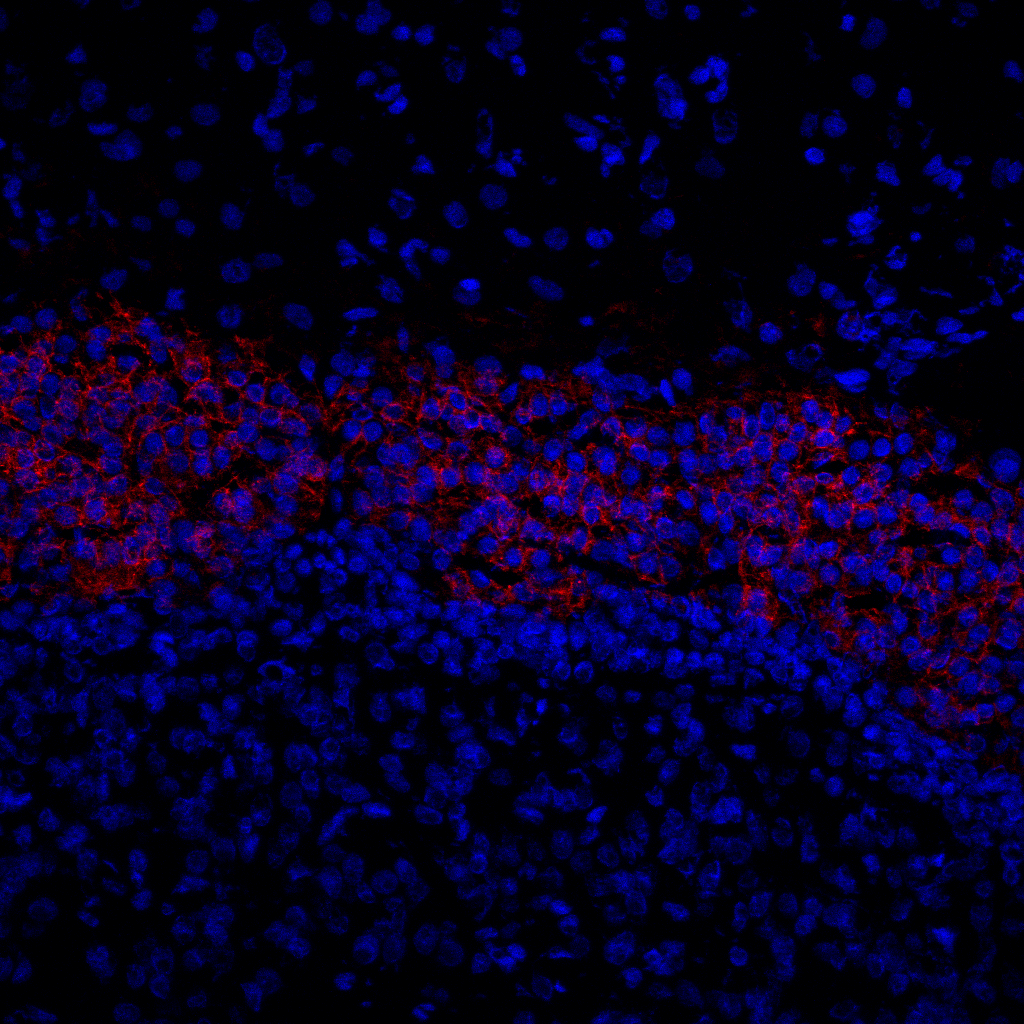

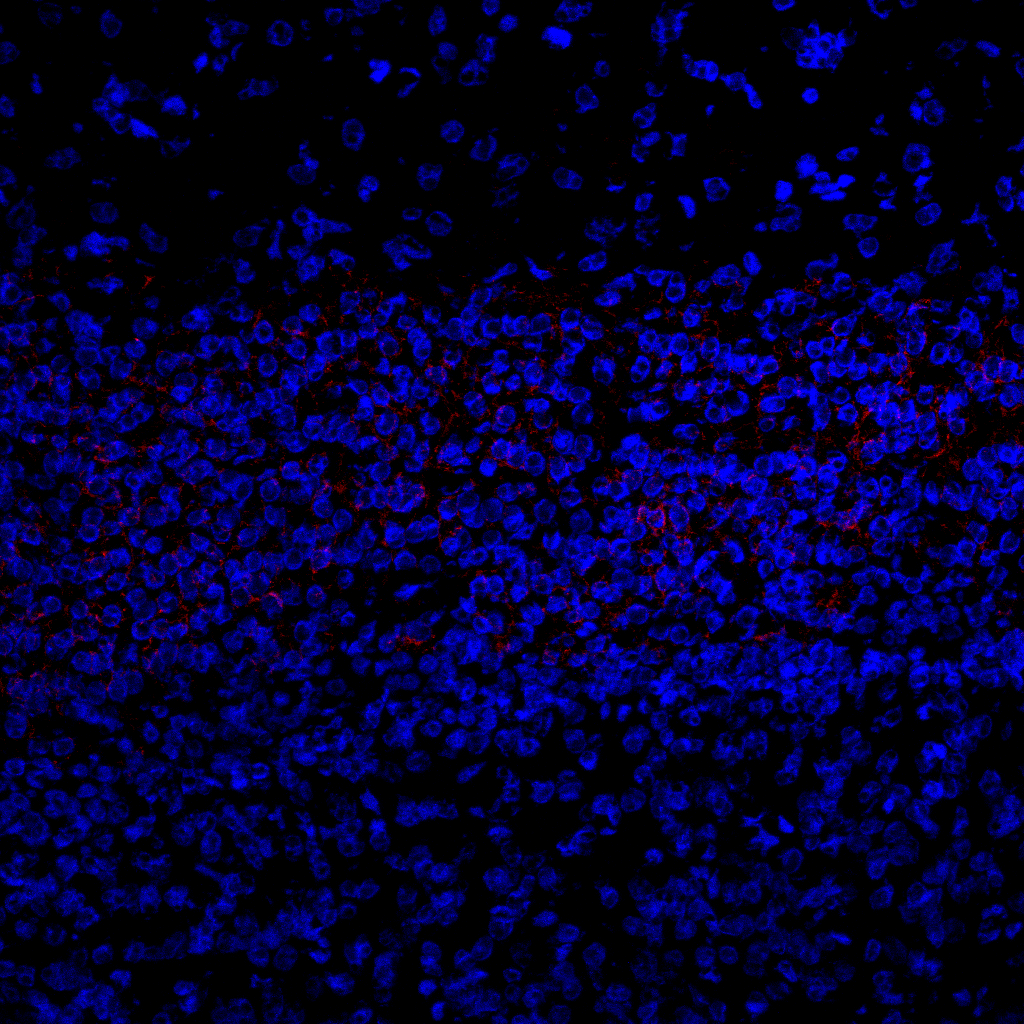


Immunofluorescence anti-Nanog 1:800

Immunofluorescence anti-Nanog 1:800+Nanog protein

Negative and preadsorption (Sox2 peptide, Chemicon AG980) controls were also used for immunohistochemistry in human and mouse sections.


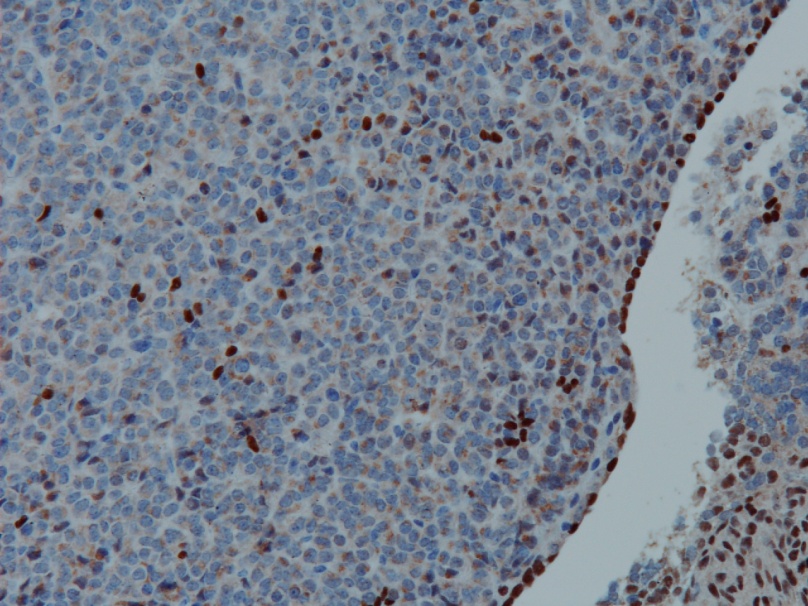

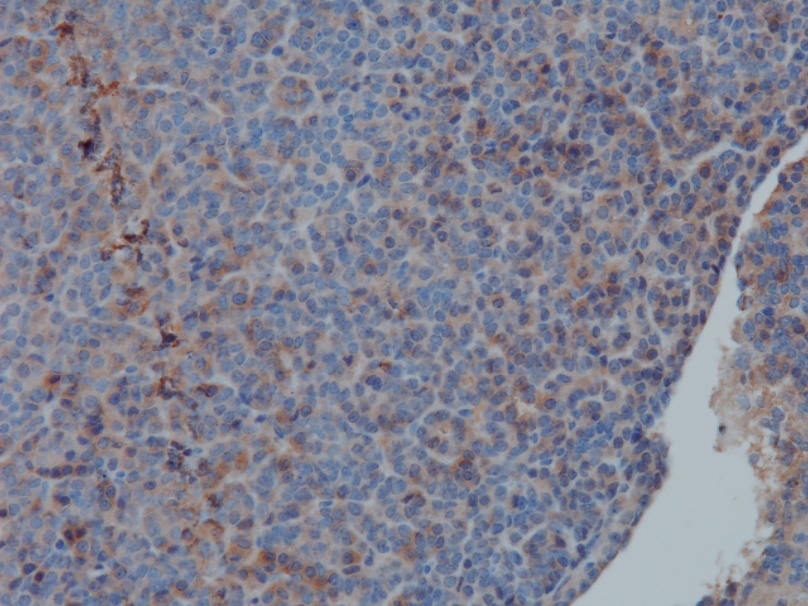


**Immunohistochemistry**

**anti-Sox2 1:300**

**Immunohistochemistry**

**anti-Sox2 1:300 + Sox2 peptide**

For a detailed list on antibodies and dilutions see Supplementary Table S1 and S2.

For immunoblotting, tissues o cells were lysed as previously described ^[4, 5]^. Shortly, after adding 1% SDS at 95 ºC during 5 minutes the lysate was diluted 1:5 with 50mM Hepes pH 7.5, 150 mM NaCl, 10% Glycerol, 1% Triton, 5 mM EGTA, 1.5 mM MgCl supplemented with 0.1 M PMSF (Sigma), 5mg/ml Aprotinine (Sigma), 2% Na_3_VO_4_ (Sigma), 0.1 M Na-pyrophosphate (Sigma), homogenized through a 20 gauge syringe and incubated for 20 minutes on ice. Lysates were spun for 5 minutes at 14000 rpm and supernatants kept at -20 ºC. 75 microg of total proteins were load in 8-15 % SDS-PAGE gels. Incubation with primary antibodies was performed overnight (see Supplementary Table S1 and S2 for dilutions) and alkaline phosphatase-conjugated secondary antibodies and the CSS system (Tropix) were used for immunodetection.

**Sorting of GFRa2+ cells.** Pituitaries from wild type male mouse were dissected and mechanically minced in PBS-1 (PBS supplemented with 2.7 mg/ml D-glucose, 10 mg/ml BSA, 0.5 mM CaCl2, 1 mM MgCl2). Fragments were further digested with 0.1% Collagenase type IA (Sigma), 0.05% (100 IU/ml) DNAse, 5 mM CaCl2 in PBS for 45 minutes with gentle agitation. After centrifuging, cells were washed for 15 minutes in PBS-2 (PBS supplemented with 2.7 mg/ml D-glucose, 10 mg/ml BSA) with gentle agitation. After this washing, cells were centrifuged and divided in two identical aliquots. The first aliquot was incubated during 45 minutes with 1: 50 anti-GFRa2 in PBS-3 (PBS supplemented with 0.5% FCS and 0.1% NaN3); the second aliquot was maintained in the same volume of PBS-3 with rabbit Ig, without antibody. After the incubation, and two further washings in PBS-3, both aliquots were incubated during 45 minutes, in the dark and with gentle agitation, with 1:50 chicken anti-rabbit Alexa 488 (Molecular Probes, A21441) in PBS-3. After two last washes in PBS-3, cells were analyzed in a FACScanto (Becton Dickinson).

**Isolation and culture of GFRa2+ cells.** Freshly isolated cell suspensions were prepared from male rat or mouse pituitaries using magnetic activated cell sorting (MACS; Miltenyi) or a fluorescence-activated cell sorter (FACS; FACSAria, Becton-Dickinson). Murine AP cells were dissected from NP and IL, washed in DMEM (Sigma) and incubated with 0.4% Collagenase type IA (Sigma), 0.05% (100 IU/ml) DNAse I (Sigma) (without/with 0.5% Trypsin, Sigma) in DMEM supplemented with 20% horse serum (HS, Gibco) in the incubator; mechanical dispersion was performed each 5 minutes. After 20 minutes, dispersed cells were washed twice in 20 % HS-DMEM and incubated with rabbit anti-GFRa2 antibody for 30 minutes at room temperature. After this incubation, 1 ml of 0.5% BSA (Sigma), 2mM EDTA in PBS (pH: 7) was added and cells were washed and resuspended in 200 μl of the same buffer plus 50 ml of anti-rabbit Microbeads (Miltenyi) for 15 minutes at 4ºC. After washing, cell-beads suspension was flowed through an MS Column (Miltenyi) attached to the magnetic separator (MiniMACS), with two 500 μl washings (GFRa2- cells). GFRa2+ cells were eluted in 1 ml of media and cultured as follows (all the experiments with spheroids shown were performed with rat cells, although many have been reproduced in mouse cells, data not shown):

1. **As spheroids:** GFRa2+ cells were eluted in 1 ml of **SpherM:** 1x N2 (Invitrogen), 1x B27 (Invitrogen), 100 IU/ml penicillin, 100 μg/ml streptomycin, and 2.5 μg/ml amphotericin-B in 1:1 DMEM/F12 (vol/vol) medium. 2500 cells were cultured in a p35 dish. After 5-7 days spheroids were either video-recorded or fixed for further immunofluorescence or induced to differentiate. For the BrdU-uptake experiments, 10 microM BrdU (Sigma) was added from the beginning, but a toxic effect was seen with longer treatments than 5 days; to evaluate the % of cell division in spheroids of different days BrdU was added for the last 12 hours of incubation before fixation. To study clonality, e.g. that the spheroids originated from a single cell, cells were also seeded in lower dilutions 20-18 cells/ml in 24-well dishes, and further diluted 1:2 in the following columns up to 0,5 cells/ml. After five days all the spheroids were photographed (Supplementary Figure 3A).

To induce differentiation, every individual spheroid was carefully pipetted under the microscope at day 5, isolated and placed in Collagen type IV or poly-L-lysine treated Cultureslides (BD) in 10 % FCS-SpherM. The following day, the medium was replaced during 15 days by one of the following differentiation media (**DifM**):

-**DifM1: 1:1 SSM+ conditioned medium from the rat-pituitary cell line GH4C1 (vol/vol)** supplemented with antibiotics. The **SSM** or Semisynthetic medium has been previously described by our group for primary pituitary culture ^[4-6]^ [6,5:3,5 DMEM/Ham‘s F12 medium (vol/vol) supplemented with (Sigma): (per liter) BSA (2 g), HEPES (2.38 g), hydrocortisone (143 μg), T3 (0.4 μg), Transferrin (10 mg), Glucagon (10 ng), Epidermal growth factor (0.1 μg), and Fibroblast growth factor (0.2 μg)]. The **conditioned medium from the GH4C1** was obtained by culturing the cell-line in DMEM+10%FCS; after 6 days without change, the medium was collected, filtered and frozen in aliquots.

**-DifM 2: 1: 1 DMEM/Ham’s F12 (1:1), 1x N2 + conditioned medium from the rat-pituitary cell line GH4C1 (vol/vol)** supplemented with antibiotics.

**-DifM 3: 1x N2, 1% FBS in SSM** supplemented with antibiotics.

**-DifM 4: 1x B27, 0,5 ng/ml FGF, 10-9 M GHRH, 10-9 M Ghrelin, 10-9M Somatostatin, 10-9M Hydrocortisone, 5 microg/ml Transferrin, 10 microg/ml Insulin in 1:1 DMEM/Ham’s F12 (vol/vol)** supplemented with antibiotics.

**-DifM5: 1:1 SSM+ conditioned medium from the rat-pituitary cell line GH3 (vol/vol)** supplemented with antibiotics. The **conditioned medium from the GH3** was obtained by culturing the cell-line in DMEM+2,5%FCS+15% Horse Serum; after 6 days without change, the medium was collected, filtered and frozen in aliquots.

1. **As attached cell culture:** GFRa2+ were eluted in 1 ml of **MEFM** and seeded in gelatin-coated wells [**MEFM: 1:1 StemM + conditioned medium from MEFs (vol/vol).** **StemM** (Millipore): 1% non-essential aminoacids, 2mM Glutamine, 0,1mM b-mercaptoethanol, 20% serum-KO replacement, 80% KO-DMEM supplemented with antibiotics. **Conditioned medium from MEF:** mouse embryonic fibroblasts were passaged in DMEM containing 20% FBS and treated with mytomicin. To obtain Conditioned medium, they were incubated in StemM (1% non-essential aminoacids, 2mM Glutamine, 0,1 mM b-mercaptoethanol 20% serum-KO replacement, 80% KO-DMEM supplemented with antibiotics); after 6 days without change, the medium was collected, filtered and frozen in aliquots.

The cells were passed every 25 days but we observed that many cells differentiated on the dish in these conditions.

1. **As floating spheres:** When cells were cultured in MEFM plus 1000 U/ml Esgro (Chemicon) (**MEFM-E**) they did not attach to the coated dish nor differentiate; they grew as groups of cells floating in the medium. They grew slowly but steady and were passed every 25 days, by centrifugation, pipet-dispersion and dilution 1:3.
2. **As embryonic-like colonies:** MEF fibroblasts were treated with mitomycin as described and frozen in aliquots. When required, an aliquot was thawed and fibroblast seeded as a monolayer. The next day, GFRa2+ purified cells were eluted in **StemM** and overlaid on top of the MEF monolayer. Two-week later colonies were formed. Attached or floating Colonies were routinely passed every 25 days with the help of Accutase (Chemicon).

Immunofluorescence of spheroids was performed pipetting them on top of 8 μm inserts (Millipore) and fixing them with 70 % Ethanol at room temperature during 30 minutes, plus 4M HCL during 20 minutes (BrdU labeling) or with -20 ºC Methanol for 5 minutes for the other antibodies before proceeding as above. Differentiated cells were fixed in Methanol (hormones) or in 4 % paraformaldehyde for 20 minutes (hormones, Tubulin-beta-III, NF). (See below for antibodies and dilutions).

**Confocal quantitative telomere FISH (Telomapping).** For telomapping, paraffin-embedded tissue sections were hybridized with a PNA-tel Cy3-labelled probe and telomere length was determined as described ^[7]^. Slides were deparaffinized in three xylene washes (3 minutes each), then treated for 3 minutes with a 100, 95 and 70% ethanol series, followed by telomere Q-FISH protocol performed as described ^[8]^. DAPI, Cy3 signals were acquired simultaneously into separate channels using a confocal ultraspectral microscope (Leica TCS-SP2-A-OBS-UV) using a PL APO 20x/0.70 PH2 as lens with Leica LCS software and maximum projections from image stacks (10 sections at steps 1.0 microM) were generated for image quantification. The DPSS-561 laser (Cy3 laser) was hold at a constant intensity to capture all the mouse tissues images. Highthoughput quantitative image analysis was performed on confocal images using the Metamorph platform (version 6.3r6; Molecular Devices, Union City, CA). The DAPI image was used to define the nuclear area and the Cy3 image to quantify of telomere fluorescence. In all cases, background noise was subtracted from each image prior to making qualitative measurements. The DAPI images were signal-intensity thresholded, segmented and converted to 1-bit binary image. The binary DAPI mask was applied to the matching Cy3 to obtain a combined image with telomere fluorescence information for each nucleus. Cy3 fluorescence intensity (telomere fluorescence) was measured as “average gray values” (total gray value/nuclei area) units (arbitrary units of fluorescence). These "average telomere fluorescence" values always represent the average Cy3 pixel intensity for the total nuclear area, and not the average value of individual telomere spot intensities, therefore ruling out that differences in nuclear size may influence telomere length measurements. A code of four colors was used to classify the nuclei according to their average telomere fluorescence. Finally, telomere fluorescence values for each histological region were exported to Excel and the frequency histograms were generated.

**Immunolabeled cell counting on paraffin sections**

Mouse pituitaries were embedded in paraffin in either sagittal or coronal orientation. A Hematoxylin staining was performed in sections every 50 microM to detect the biggest section per pituitary. Immunohistochemistry was performed in three sections around the middle separated by at least 20 microM. A minimum of 3 pituitaries per orientation and 250 cells per mice were scored (at least two males and two females). The number of cells per area was counted in sections. On the other hand, the volume of the pituitary was calculated using the following equation (volume of an ellipsoid):

Pituitary volume = (Major axis × Minor axis^2^)/2

(as mean among four mice per group).

**qRT-PCR primers and conditions.** Total RNA from male rat pituitaries was extracted with RNeasy Mini Kit (Quiagen). 1 μg RNA was reverse-transcribed using the standard protocol for MMLV (Invitrogen) in 20 μl reaction. 3 μl of the reaction were amplified with Cybergreen PCR mix (Roche) in a 7300 TaqMan (Applied), using the following conditions: 95ºC 2”, 60ºC 15”, 72ºC 15”. A list of the Oligonucleotides used is shown in Supplementary table S3.

**References**

1. Japon MA, Urbano AG, Saez C, Segura DI, Cerro AL, et al. (2002) Glial-derived neurotropic factor and RET gene expression in normal human anterior pituitary cell types and in pituitary tumors. J Clin Endocrinol Metab 87(4): 1879-1884.

2. Urbano AG, Suarez-Penaranda JM, Dieguez C, Alvarez CV. (2000) GDNF and RET-gene expression in anterior pituitary-cell types. Endocrinology 141(5): 1893-1896.

3. Krylyshkina O, Chen J, Mebis L, Denef C, Vankelecom H. (2005) Nestin-immunoreactive cells in rat pituitary are neither hormonal nor typical folliculo-stellate cells. Endocrinology 146(5): 2376-2387.

4. Canibano C, Rodriguez NL, Saez C, Tovar S, Garcia-Lavandeira M, et al. (2007) The dependence receptor ret induces apoptosis in somatotrophs through a pit-1/p53 pathway, preventing tumor growth. EMBO J 26(8): 2015-2028.

5. Garcia A, Alvarez CV, Smith RG, Dieguez C. (2001) Regulation of pit-1 expression by ghrelin and GHRP-6 through the GH secretagogue receptor. Mol Endocrinol 15(9): 1484-1495.

6. Coya R, Alvarez CV, Perez F, Gianzo C, Dieguez C. (1999) Effects of TGF-beta1 on prolactin synthesis and secretion: An in-vitro study. J Neuroendocrinol 11(5): 351-360.

7. Flores I, Canela A, Vera E, Tejera A, Cotsarelis G, et al. (2008) The longest telomeres: A general signature of adult stem cell compartments. Genes Dev 22: 654-667.

8. Samper E, Goytisolo FA, Slijepcevic P, van Buul PP, Blasco MA. (2000) Mammalian Ku86 protein prevents telomeric fusions independently of the length of TTAGGG repeats and the G-strand overhang. EMBO Rep 1(3): 244-252.
