# Supplementary material for: A GRFa2/Prop1/Stem (GPS) Cell Niche in the Pituitary
Source: PLoS One. 2009 Mar 13;4(3):e4815. doi: 10.1371/journal.pone.0004815 (PMC2654029; doi:10.1371/journal.pone.0004815)
Supplement: Table S1 — List of Antibodies and dilutions. Primary Antibodies (0.06 MB DOC) [file pone.0004815.s013.doc]

**Supplementary Table S**1. List of Antibodies and dilutions. Primary Antibodies

| **Protein** | **Species** | **Catalog Nº** | **Company** | **Dilution** | | | |
| --- | --- | --- | --- | --- | --- | --- | --- |
|  |  |  |  | **Tissue** | **Spheroid** | **Differentiation** | **WB** |
| GFRa2 | Rabbit | AB5141 | Chemicon | 1:500 | 1:50 | 1:50 | 1:600 |
| GH | Rabbit | AFP-C11981A | NIDDK | 1:800 | 1:300 | 1:300 | 1:1000 |
| GH | Guinea pig | AFP 222 387 790 | NIDDK | 1:800 |  |  |  |
| ACTH | Mouse | M3501 | Dako | 1:100 |  | 1:50 |  |
| PRL | Mouse | E30610M | Biodesign | 1:200 | 1:100 | 1:100 | 1:1000 |
| beta-TSH | Goat | sc-7813 | Santa Cruz B | 1:200 |  |  |  |
| beta-TSH | Rabbit | AFP-1274789 | NIDDK |  |  | 1:50 |  |
| beta-FSH | Mouse | 18-0020 | Zymed Laboratories | 1: 75 |  | 1:50 |  |
| beta-LH | Mouse | MS-9078-P | Neo Markers | 1:250 |  |  |  |
| Cytokeratins | Mouse | NCL-C11 | NovoCastra | 1:45 |  |  |  |
| E-Cadherin | Mouse | C20820-050 | BD biosciences | 1:75 | 1:50 |  |  |
| b-Catenin(IF) | Mouse | 05-665 | Upstate | 1:300 | 1:50 |  |  |
| b-Catenin(IHQ) | Rabbit | sc-1496 | Santa Cruz B | 1:200 |  |  |  |
| Oct4 | Mouse | MAB4305 | Chemicon | 1:100 | 1:50 |  |  |
| SSEA-4 | Mouse | MC-813-70 | Hybridoma bank | 1:50 |  |  |  |
| Nanog | Rabbit | Ab5731 | Chemicon | 1:800 |  |  |  |
| Prop1 | Guinea pig | Against GST-Prop_155-230_  and His-Prop_155-230_    C-terminal:  bCA | in house A.K. Ryan | 1:250 | 1:50 | 1:50 | 1:500 |
| S100 (rat) | Rabbit | RTU-RTUS100p | Novocastra | Commercially pre-diluted |  |  |  |
| S100  (mouse, human) | Rabbit | Z0311 | Dako | 1:100 (mouse)  1:2000 (human) |  |  |  |
| Vimentin (rat) | Rabbit | (H-84): sc-5565 | Santa Cruz B | 1:200 |  |  |  |
| Vimentin (mouse) | Guinea Pig | RDI-PROGP53 | Fitzgerald | 1:25  (Prot K) |  |  |  |
| Vimentin (human) | Mouse | | clone V9 | M0725 | | --- | --- | | Dako | 1:500 (Citrate) |  |  |  |
| Nestin | Mouse | Rat-401 | Hybridoma bank | 1:100 |  |  |  |
| Ki67(rat) | Mouse | M7248 | Dako | 1:250 |  |  |  |
| Ki67(mouse) | Mouse | 000310Q10(SP6) | Master D | 1:200 |  |  |  |
| Sox2 | Mouse | MAB4343 | Chemicon | 1:100 |  |  |  |
| Sox2 | Rabbit | AB5603 | Chemicon | 1:300 (IHQ)  1:500 (IF) |  |  |  |
| Sox9 | Rabbit | AB5535 | Chemicon | 1:200 (IHQ)  1:500 (IF) |  |  |  |
| Sox4 | Rabbit | S7318 | Sigma | 1:50 |  |  |  |
| NTN | Goat | AF477 | R&D systems | 1:100 |  |  | 1:500 |
| Ret | Goat | sc-1290 | Santa Cruz B | 1:100 |  |  |  |
| Pit1 | Rabbit | sc-442 | Santa Cruz | 1:300 |  |  |  |
| beta-Tubulin  isotype III | Mouse | T8660 | Sigma |  |  | 1:100 |  |
| Neurofilament  (PAN) | Mouse | FNP7,DA2,RmdO2011 | Zymed |  |  | Commercially  pre-diluted |  |
