# Supplementary material for: A GRFa2/Prop1/Stem (GPS) Cell Niche in the Pituitary
Source: PLoS One. 2009 Mar 13;4(3):e4815. doi: 10.1371/journal.pone.0004815 (PMC2654029; doi:10.1371/journal.pone.0004815)
Supplement: Table S2 — List of secondary antibodies and related reagents. (0.04 MB DOC) [file pone.0004815.s014.doc]

**Supplementary Table S2. List of secondary antibodies and related reagents.**

| **Secondary antibody** | **Labeling** | **Catalog number** | **Company** | **Dilution** | | | |
| --- | --- | --- | --- | --- | --- | --- | --- |
|  |  |  |  | **Tissue** | **Spheroid** | **Differentiation** | **WB** |
| Rabbit anti-Mouse | Cy^TM^2 | 315-226-047 | Jackson InmunoResearch | 1:1000 | 1:400 | 1:400 |  |
| Goat anti-Rabbit | Cy^TM^3 | 111-166-047 | Jackson InmunoResearch | 1:1000 | 1:600 | 1:600 |  |
| Donkey anti-Goat | Alexa Fluor 488 | A-11055 | Molecular Probes | 1:250 |  |  |  |
| Goat anti-Guinea Pig | Alexa Fluor 488 | A-11073 | Molecular Probes | 1:1500 | 1:600 |  |  |
| Goat anti-rabbit | Botinylated | BA-100 | Vector Labs | 1:200 |  |  |  |
| Goat anti-mouse | Biotinilated | BA-200 | Vector Labs | 1:200 |  |  |  |
| Goat anti-Rabbit | Alcaline phosphatase phosphatase | AC31RL | Tropix |  |  |  | 1:5000 |
| Goat anti-Mouse | Alcaline phosphatase phosphatase | AC32ML | Tropix |  |  |  | 1:5000 |
| Protein A | HRP | NA9120V | Amersham |  |  |  | 1:5000 |
