# Supplementary material for: A GRFa2/Prop1/Stem (GPS) Cell Niche in the Pituitary
Source: PLoS One. 2009 Mar 13;4(3):e4815. doi: 10.1371/journal.pone.0004815 (PMC2654029; doi:10.1371/journal.pone.0004815)
Supplement: Table S3 — Oligonucleotides used to analyze gene expression by RT-PCR. (0.03 MB DOC) [file pone.0004815.s015.doc]

**Supplementary Table S**3. Oligonucleotides used to analyze gene expression by RT-PCR.

| **Genes** | **Forward (F) and Reverse (R) Primers.** | **Fragment (bp)** |
| --- | --- | --- |
| GRFa2 | F-5´TTCAGGCTCGCTTCAATCTT 3´  R-5´AACTGCAAGAAGCTTCGCTC 3´ | 122 |
| Oct4 | F-5´CAAGTTGGCGTGGAGACTCTGC 3´  R-5´AGACAACCATCTGCCGCTTCG 3´ | 299 |
| Prop1 | F-5´GACAGCTGGAGTCAGCCTTT 3´  R-5´GCAAGAGCGGTCACTACTCC 3´ | 153 |
| GH | F-5´GCAGAGAACTGACATGGAATTG 3´  R-5´ GATGACGCTCTGCTCAAAA 3´ | 269 |
| Ret | F-5´ CATCAAGTTGTACGGGGCTT 3´  R-5´ TGCCTCCACTGCTCACATAG 3´ | 134 |
| Pit1 | F-5´TTCCAGACCACACCCTGAGT 3´  R-5´ACTTTTCCGCCTGAGTTCCT 3´ | 190 |
| Hprt | F-5´CAGTCCCAGCGTCGTATT 3´  R-5´AGCAAGTCTTTCAGTCCTGTC 3´ | 139 |
